# Papain-Mediated Conjugation of Peptide Nucleic Acids to Delivery Peptides: A Density Functional Theory/Molecular Mechanics Metadynamics Study in Aqueous and Organic Solvent

**DOI:** 10.1021/acs.jpcb.4c02294

**Published:** 2024-07-25

**Authors:** Ricardo D. González, Alexandra T. P. Carvalho

**Affiliations:** † CNC-UC − Center for Neuroscience and Cell Biology, University of Coimbra, Coimbra 3004-504, Portugal; ‡ CIBB − Center for Innovative Biomedicine and Biotechnology, University of Coimbra, Coimbra 3004-504, Portugal; § Institute for Interdisciplinary Research, Doctoral Programme in Experimental Biology and Biomedicine (PDBEB), University of Coimbra, Coimbra 3030-789, Portugal; ∥ Almac Sciences, Department of Biocatalysis and Isotope Chemistry, Almac House, 20 Seagoe Industrial Estate ,Craigavon, Northern Ireland BT63 5QD, United Kingdom

## Abstract

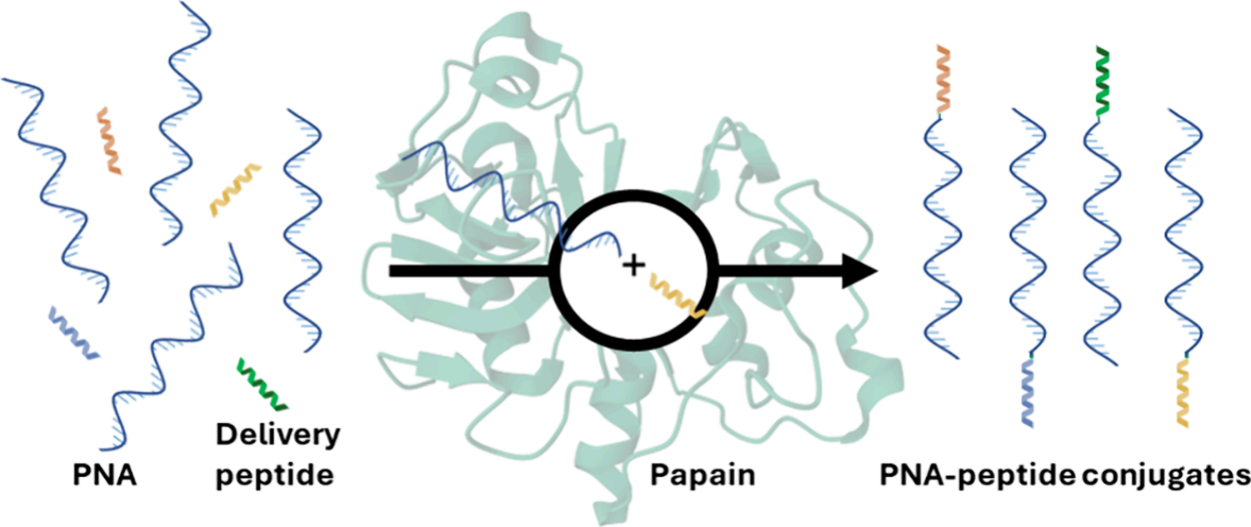

Enzymatic peptide synthesis is a powerful alternative to solid-phase methods, as enzymes can have high regio- and stereoselectivity and high yield and require mild reaction conditions. This is beneficial in formulation research due to the rise of nucleic acid therapies. Peptide nucleic acids (PNAs) have a high affinity toward DNA and RNA, and their solubility and cellular delivery can be improved via conjugation to peptides. Here, we designed and assessed the viability of the papain enzyme to conjugate four PNA-peptide models in water and an organic solvent using QM/MM metadynamics. We found that the reactions in water yield better results, where three conjugates could potentially be synthesized by the enzyme, with the first transition state as the rate-limiting step, with an associated energy of 14.53 kcal mol^–1^, although with a slight endergonic profile. The results highlight the importance of considering the enzyme pockets and different substrate acceptivities and contribute to developing greener, direct, and precise synthetic routes for nucleic acid–based therapies. By exploring the enzyme’s potential in conjunction with chemical synthesis, current protocols can be simplified for the synthesis of longer nucleic acids and peptide sequences (and, by extension, proteins) from smaller oligo or peptide blocks.

## 1 Introduction

**Figure 1:**
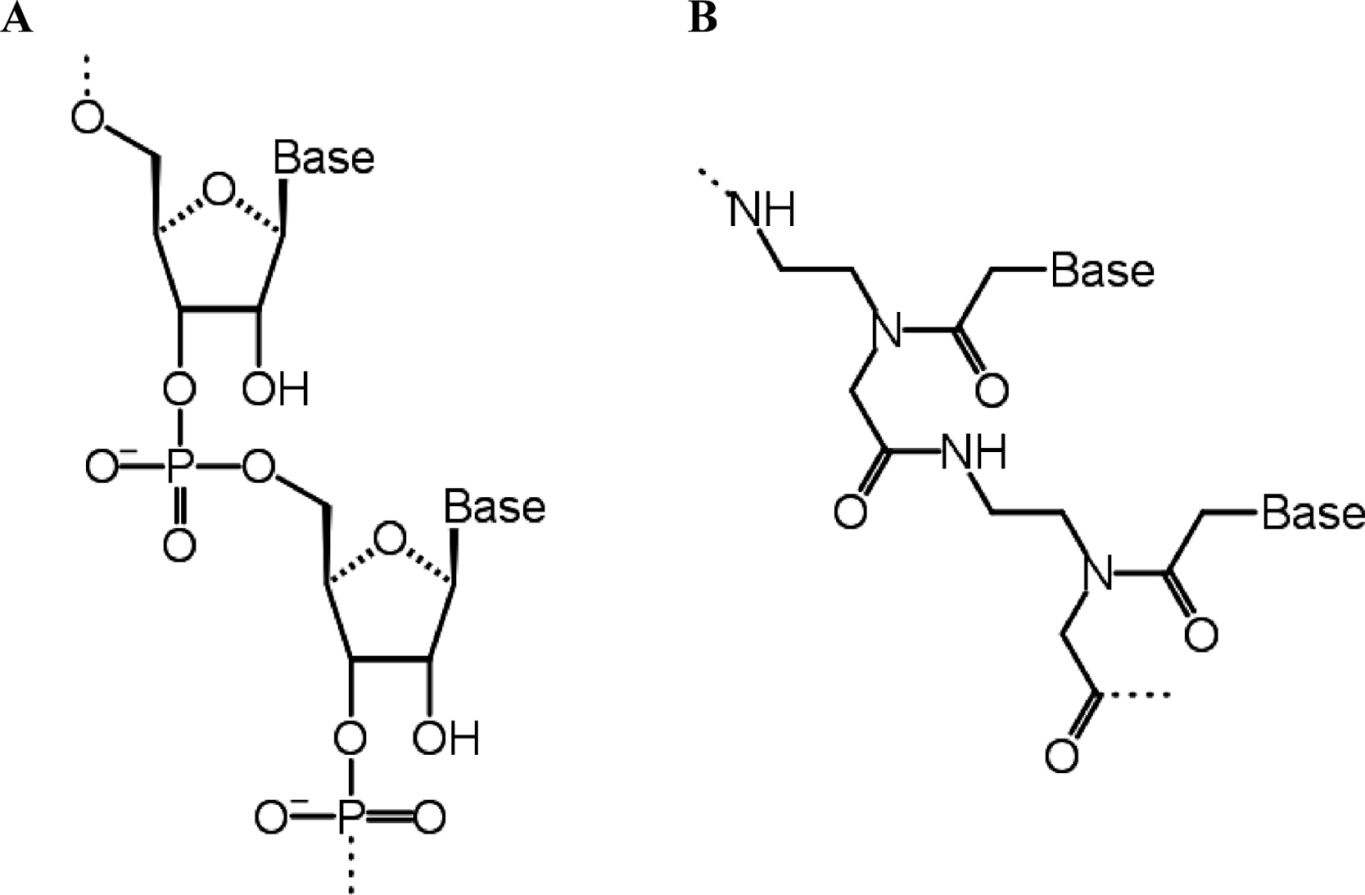
Schematic model of (A) RNA and (B) PNA oligomers. The RNA’s phosphodiester backbone is replaced by a polyamide backbone in the PNA.

Peptide nucleic acids (PNAs), first described in 1991 by Nielsen and colleagues,^1^ are synthetic nucleic acid analogs that lack the conventional phosphodiester backbone (Figure 1). They have a neutral-charged polyamide backbone (*N*-2-(aminoethyl)-glycine)) that allows them to bind to nucleic acids with greater affinity than natural nucleic acids in a sequence-specific manner, acting as an antisense oligonucleotide (ASO) via steric blockage.^2^ Contrary to other ASOs, the PNA backbone makes it resistant to nuclease and protease-mediated degradation by not recruiting the RNase-H or RNA-induced silencing complex, which makes PNA action concentration-dependent. Hence, their intracellular delivery must be maximized so that there is an optimized concentration at the target site, which may be accomplished by conjugating them with delivery moieties, such as cell-penetrating peptides (CPPs), which have been previously successful in delivering PNAs to cells.^3−5^

CPPs are small peptides (6 to ca. 30 amino acids) capable of transporting cargo across the cellular membranes.^6^ They have been used in conjunction with other delivery methods, such as nanoparticles, polymeric micelles, or liposomes, allowing for enhanced endosomal escape via conjugation on the surface of those structures.^7,8^ Because PNAs are more stable than other ASOs, protection of the active molecule may not be necessary, and they can be delivered using simpler delivery methods (such as CPPs), granting for easier targeted therapy design.

Nevertheless, peptide synthesis usually relies on the solid-phase synthesis (SPPS) method, which is good for small peptides but is harder to apply to longer sequences (with companies only producing peptides with up to 40 residues), since as the sequence length increases, impurities do also, making the final product more difficult to obtain and isolate.^9^ Furthermore, the method requires multiple protection and deprotection steps as well as the use of toxic chemicals.^10,11^

Additionally, PNA synthesis through SPPS displays poor reaction yields,^12^ even for short sequences, especially for purine-rich oligos, due to the steric properties of those nucleobases,^13^ while PNA’s poor water solubility induced by the neutral charge character allows them to aggregate, leading to poor intracellular uptake.^14^ Even though this drawback can be overcome when conjugating them with delivery peptides, length constraints (a longer PNA sequence would improve selectivity toward the targeted sequence) and complexity limit the use of the SPPS method, highlighting the need for more effective methods for PNA and PNA conjugates synthesis. It is here that chemoenzymatic methods have the upper hand, with enzymes presenting key benefits such as regio- and stereoselectivity, high yield, absence of the requirement for side chain protection, and mild reaction conditions.^15^

**Scheme 1:**
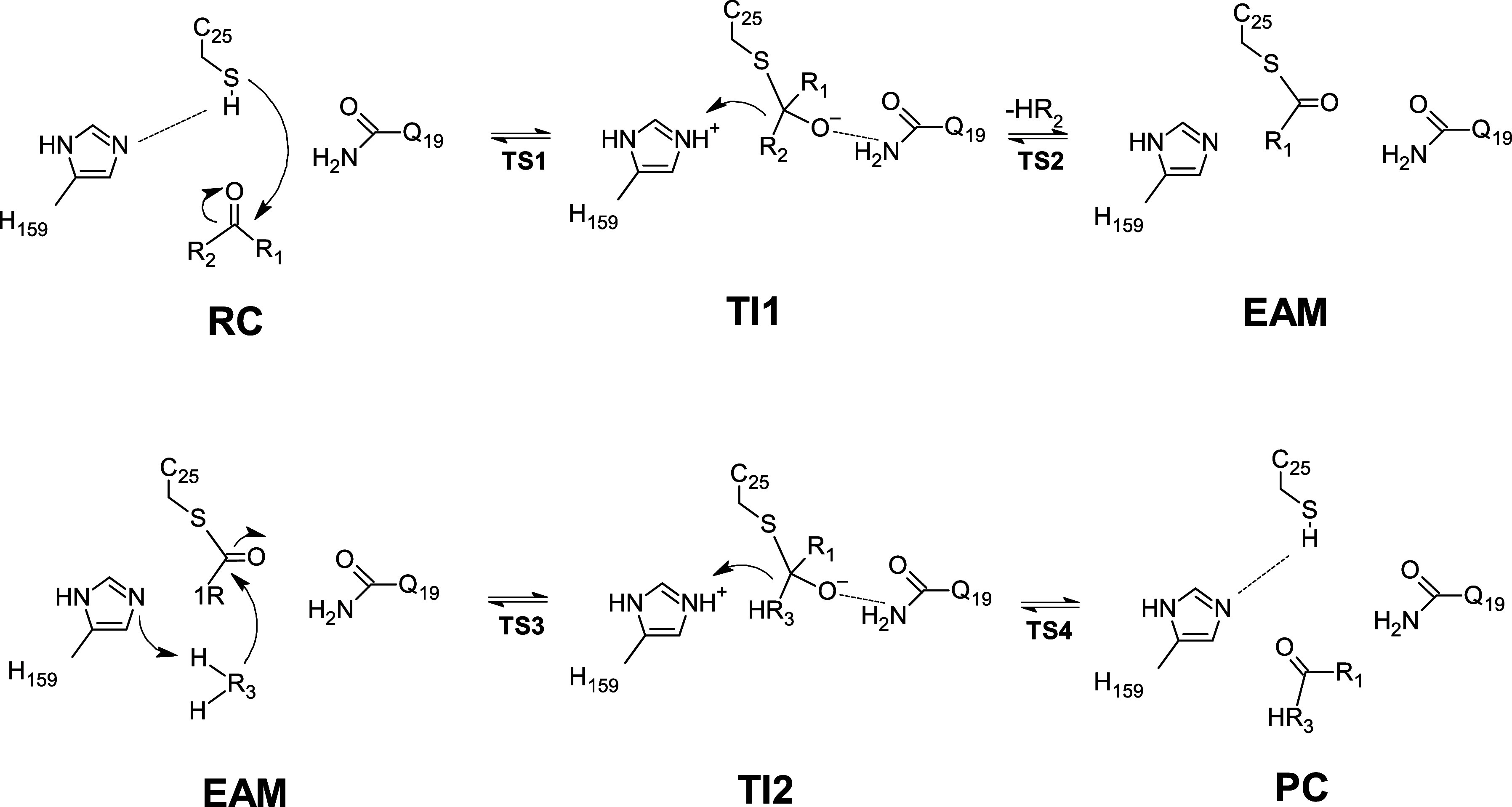
General Catalytic Cycle for the Papain Enzyme **R**_**1**_ = α-*N*-benzoyl-L-citrulline or PNA, **R**_**2**_ = methyl ester or ethanol, **R**_**3**_ = oxygen (water molecule) or peptide.

Although the cleavage of peptide bonds by proteases is widely known, chemoenzymatic synthesis makes use of the fact that, in some circumstances, proteases can catalyze the formation of peptide bonds.^15−17^ The cysteine protease papain (EC 3.4.22.2) has been widely explored in chemoenzymatic peptide synthesis and thoroughly investigated utilizing a variety of substrates, from milk and hemoglobin to synthetic peptides, with an increased interest in a wide range of industrial settings, mainly in meat tenderization, brewing, and the textile industry.^15,18−22^ In terms of enzymatic activity, papain possesses a broad-spectrum endopeptidase activity at pH 5–8 and an optimum temperature of 65 °C.^23^ Papain’s active site is located at the interface of the L- and R-domains that form a V-shaped cleft and is composed of cysteine (C25), histidine (H159), asparagine (N175), and glutamine (Q19) residues, which are conserved in all papain-like proteases.^24^ N175 stabilizes the H159 (with a similar role as the aspartic acid in the catalytic triad of serine proteases) but is not required for catalysis,^25^ orienting H159’s imidazolium ring toward the thiol group of C25, leading to deprotonation and forming the ion-pair dyad. The glutamine residue (Q19) helps form an oxyanion hole along with the amide group of C25, stabilizing the tetrahedral intermediate (**TI1**) negative charge (Scheme 1).^24^

Papain’s mechanism has been reported both as concerted and stepwise for different substrates.^26−29^ It comprises the formation of the enzyme-activated monomer (**EAM**) structure, resulting in the alcohol release after nucleophilic attack of C25 to the substrate’s ester group. The product complex (**PC**) is formed during the deacylation step, via nucleophilic attack of a water molecule (or peptide, in this study), which produces the tetrahedral intermediate 2 (**TI2**). Finally, the proton transfer from H159 to C25 leads to **PC** formation (Scheme 1).

Many computational studies on the hydrolysis reaction of peptide bonds by cysteine proteases, including papain, have been conducted, including the exploration of peptide bond formation via aminolysis.^19,30−32^ For instance, Gimenez-Dejoz et al.^19^ have explored the acceptance of both L- and D-amino acids (alanine ethyl ester) in polymerization reactions performing adaptively biased QM/MM MD calculations with the DFT B3LYP/6-31G* basis set for the QM atoms. They found that for the acylation step, both reactions occur in a concerted mechanism, but in aminolysis, it occurs in a stepwise mechanism for the L-amino acid with a lower energy barrier (12 kcal mol^–1^) compared to the D-amino acid, which is concerted (30 kcal mol^–1^) and prefers the hydrolysis reaction. Yet, to the best of our knowledge, no research has been conducted using papain as an intermediary for the synthesis of nucleic acid–based therapies.

Thus, we aimed to explore the feasibility of (peptide) nucleic acid-based conjugate enzymatic synthesis as an approach for a greener production of advanced therapies, helping with the drawbacks of SPPS methods and the need for previous chemical modification of the peptides to be ligated (e.g., the use of Sortase A to ligate PNA to a CPP via modification with the LPXTG sorting signal and polyglycine chains).^33^ First, to ensure that the results from our systems were representative of the real yields, we evaluated the hydrolysis mechanism of α-*N*-benzoyl-L-citrulline methyl ester (BCME) into α-*N*-benzoyl-L-citrulline and methanol, from which previous experimental data is available, but also due to the structural similarities with a PNA nucleobase, such as the presence of amide bonds, the peptide linkage in α-*N*-benzoyl-L-citrulline (BC), and the peptidic nature of the PNA backbone as well as the extended backbones with aromatic side chains, which could create parallels in how these molecules interact with others, for instance via hydrogen bonding or stacking interactions (Supporting Information (SI), Figure S1). After that, studies regarding the synthesis of a PNA conjugated to different delivery peptides were completed via QM/MM metadynamics simulations with the B3LYP/GPW level of theory. We have selected four peptides (Table 1): the well-known CPP TAT^34^ and the three best ranked peptides from our previous work, designed from a deep learning method, from which Pep5 displayed better internalization than TAT in *in vitro* studies with HeLa cells at early incubation time points.^35^

**Table 1:** Sequence and Length of the Delivery Peptides and the Nucleic Acid^a^

	sequence	length (aa/bp)
TAT	**GRK**KRRQ***RRRPQ***	12
Pep5	**RRK**FFRLRWPWLWRRRRRRCWNRIQ***KRFGGA***	31
Pep7	**RGL**LLPSLRLRVRRRRRRR	19
Pep10	**KWK**SFLHVVKALTKVGKAVFSGVFDMIKCKISGGC	35
PNA	NH_2_–CAUCACUACGCAACUUUAG**AU**-CO_2_CH_2_CH_3_	21

a In bold, the modeled residues used in the QM/MM metadynamics simulations in water; in bold and italic, in organic solvent.

## 2 Methods

### 2.1 Initial Setup

For the simulations in water, the substrates were modeled containing a methyl ester and an ethyl ester terminal group for BCME and PNA, respectively. The choice of ethyl ester rather than a methyl ester as in the BCME was because, in the PNA synthesis reaction, an ethyl ester is present during reaction steps.^36^ We hypothesized then that the PNA could potentially be commercialized with this terminal, which would take part in the aminolysis reaction without further terminal modifications. For the simulations in organic solvent, however, we performed the screening by using the PNA’s terminal amide, and the substrate was modeled accordingly. The initial tetrahedral intermediate (TI) structures of the enzyme–substrate were modeled from the *Carica papaya* papain (PDB ID 1PPN, monomer (enzyme active structure) with 1.6 Å resolution)^37^ crystal structure. The protonation states of histidine and cysteine residues were assigned with MolProbity^38^ and confirmed via visual inspection. The TI1 and TI2 structures were geometry optimized in Gaussian09^39^ using B3LYP^40^ with the 6-31G(d,p) basis set. Their atomic partial charges were calculated resorting to the Restrained Electrostatic Potential (RESP) methodology.^41^ The initial position of the tetrahedral structures (covalent bond of the substrate to C25) was obtained by the flexible side-chain covalent docking method^42^ and performed with the AutoDock4.2 suite of programs using the Lamarckian Genetic Algorithm (LGA).^43^ A total of 150 runs were carried out. The population was set to 300, the maximum number of generations to 27,000, and the maximum number of energy evaluations to 2,500,000.

### 2.2 Molecular Dynamics

The AMBER18^44^ software was used to perform molecular dynamics (MD) simulations, with the Amber ff14SB force field^45,46^ for both peptidic moieties (peptides and peptide nucleic acid). The initial TI1 and TI2 structures were placed within a cubic box with TIP3P water molecules (on average 80 × 72 × 61 Å), and sodium ions were added to neutralize the system.^44^ For the dynamics with organic solvent (20% methanol), TIP3P water and MeOH molecules were added. The ParmEd utility of AmberTools was used to add the Lennard-Jones parameters.^47^ An initial energy minimization and 50 ps of heating and 50 ps of density equilibration with weak restraints (2.0 kcal mol^–1^) were carried out on the systems, with temperature set to 338.15 K, prior 5 ns of constant pressure equilibration without any restraint. 50 ns production simulations were then performed for each system, at 338.15 K in an NPT ensemble using the Langevin dynamics with a collision frequency of 1 ps^–1^. Constant pressure periodic boundary conditions were imposed, with an average pressure of 1 atm. Isotropic position scaling was used to maintain pressure with a relaxation time of 2 ps, and the time step was set to 2 fs. SHAKE constraints were applied to all bonds involving hydrogen atoms.^48^ The electrostatic interactions were calculated resorting to the particle mesh Ewald (PME) method^49^ with a cutoff distance of 10.0 Å.

### 2.3 Quantum Mechanics/Molecular Mechanics Metadynamics Simulations

The Quantum Mechanics/Molecular Mechanics (QM/MM) metadynamics simulations were performed with the hybrid QM/MM scheme implemented in CP2K (v9.1).^50^ The last MD production structure of each complex was used as the initial structure for these calculations. Periodic boundary conditions were applied, and the long-range interactions were computed with the smooth particle mesh Ewald (SPME) method for nonbonded interactions, with a cutoff of 10 Å.^51^ CP2K’s QUICKSTEP and FIST methods were employed to compute the QM and MM layers forces, respectively.^50,52,53^ The B3LYP/GPW level of theory was applied for the QM region with a plane-wave cutoff of 300 Ry, combined with the Gaussian plane-wave basis set DZVP-GTH-BLYP to describe valence electrons and GTH-BLYP for the core electrons. The parameters for the MM region were taken from the starting MD simulations, where the Amber ff14SB force field^46^ was applied. The QM unit cell size and systems’ total charge were defined according to each system with a singlet spin multiplicity. The structures were equilibrated in the NVT ensemble at 338 K with the Nosé–Hoover thermostat, with an integration time-step of 1 fs for 2 ps, using the fast Gaussian expansion as electrostatic coupling scheme.^54,55^ To accelerate the Hartree–Fock exchange required for B3LYP calculations, we have resorted to the auxiliary density matrix method (ADMM).^56^

**Figure 2:**
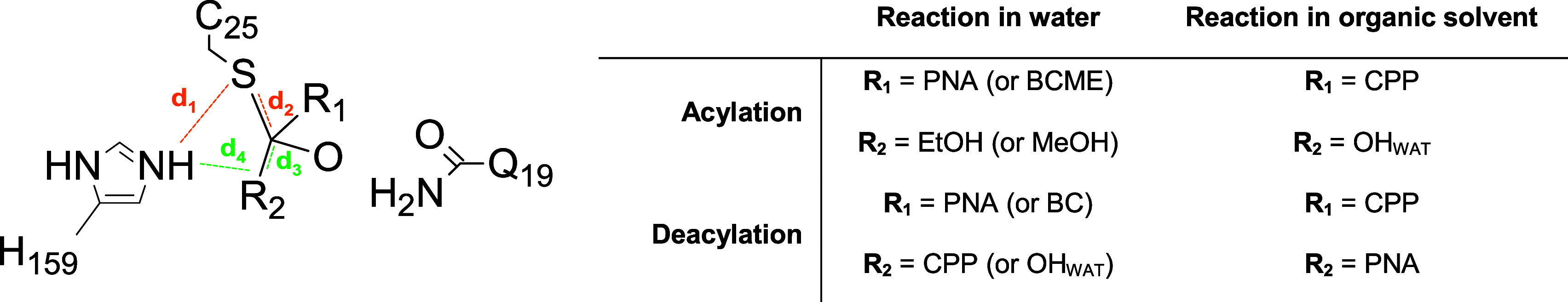
Schematic representation of the collective variables used in the QM/MM metadynamics simulations. d_1_ and d_4_ represent the proton transfer to and from H159, and d_2_ and d_3_ correspond to the C–S and C-R_2_ bond forming and breaking. Here, R_1_ is the α-*N*-benzoyl-L-citrulline or the peptide nucleic acid (in aqueous solvent) or a peptide (organic solvent), and R_2_ is an alcohol in the acylation step, a water molecule or peptide in the deacylation (in aqueous solvent), or a water in the acylation step, and the peptide nucleic acid (in organic solvent).

A set of descriptors (collective variables, CVs) was chosen for the enhanced sampling, which would be able to characterize all of the states of the simulated systems (Figure 2). A CV must be a differentiable function of the atomic coordinates. Here, we have employed a subtractive distance function (CV_1_–CV_2_), where CV_1_ = (d_1_–d_2_) and CV_2_ = (d_3_–d_4_), with d_1_ and d_4_ representing the proton transfer to and from H159, and d_2_ and d_3_ correspond to the C–S and C-R bond forming and breaking (R being an alcohol in the acylation step and a water molecule or peptide in the deacylation, in aqueous solvent simulations; or a water molecule in the acylation step and a PNA in the deacylation step, in organic solvent). Each CV was applied as a constraint and ran for up to 30 ps (or until the reaction occurs) with a 0.5 fs integration time-step. In the metadynamics technique, the Gaussians were added every 30 fs. The height of the Gaussians was defined in the 1.0–2.5 kcal mol^–1^ range, and the width of the Gaussians was 0.5–2.5 Å. In the extended Lagrangian metadynamics technique, the parameters of the collective variables were defined with a virtual particle mass of 20.0 AMU and a coupling spring constant of lambda (λ) = 0.8. The free energy landscapes (FELs) (SI Figures S2–S4) were obtained by integrating all the Gaussians used to bias the system, resorting to a tool implemented in CP2K (graph.sopt).^50^ Throughout the manuscript, we present the corresponding potential of mean force (PMF) for easier visualization of the reaction states. This is because in metadynamics when using finite-width Gaussians and atom coordination numbers as CV, as we used here, exploration of the energetic landscape may produce lower smoothness profiles, as the Gaussians quickly fill the free-energy surface, creating “artificial” shallow wells that do not correspond to real physical states. By being aware of this particular feature of the method and by carefully analyzing the produced trajectories and FELs, one can more accurately identify the relevant states of the reaction, taking into account the calculated CV function. This allows the filtering of these nonrelevant configurations that might appear while the CV function is exploring the energetic landscape.^57−59^

## 3 Results and Discussion

### 3.1 α-*N*-Benzoyl-L-citrulline methyl ester hydrolysis

The α-*N*-benzoyl-L-citrulline methyl ester (BCME) compound is a derivative of L-citrulline, which plays a key role in the urea cycle, and belongs to the class of *N*-acytaled amino acids.^60^ To evaluate the performance of QM/MM metadynamics simulations with the B3LYP/GPW level of theory implemented on CP2K, we computed the free energies associated with the hydrolysis of BCME into α-*N*-benzoyl-L-citrulline and methanol and compared the experimental values previously reported in kinetics studies for this reaction.^61,62^ To the best of our knowledge, this kind of computational study has not previously been performed on the compound. The free energy profiles can be found in SI Figure S5, along with detailed mechanistic insights on acylation (SI Figure S6) and deacylation (SI Figure S7). The reported turnover number (*k*_cat_) by Cohen and Petra^61^ is 15.54 ± 1.62 s^–1^ for the hydrolysis of BCME. According to Eyring’s equation,^63^ this corresponds to a free energy of about 15.7 kcal mol^–1^ at 25 °C. Our simulations suggest that this reaction follows a stepwise mechanism and that the rate-limiting step is the formation of **TS1** with a Δ*G*^‡^ of 13.64 kcal mol^–1^, which is in close agreement with this value (SI Figure S5).

### 3.2 Peptide Nucleic Acid-Delivery Peptide Conjugation in Water

**Figure 3:**
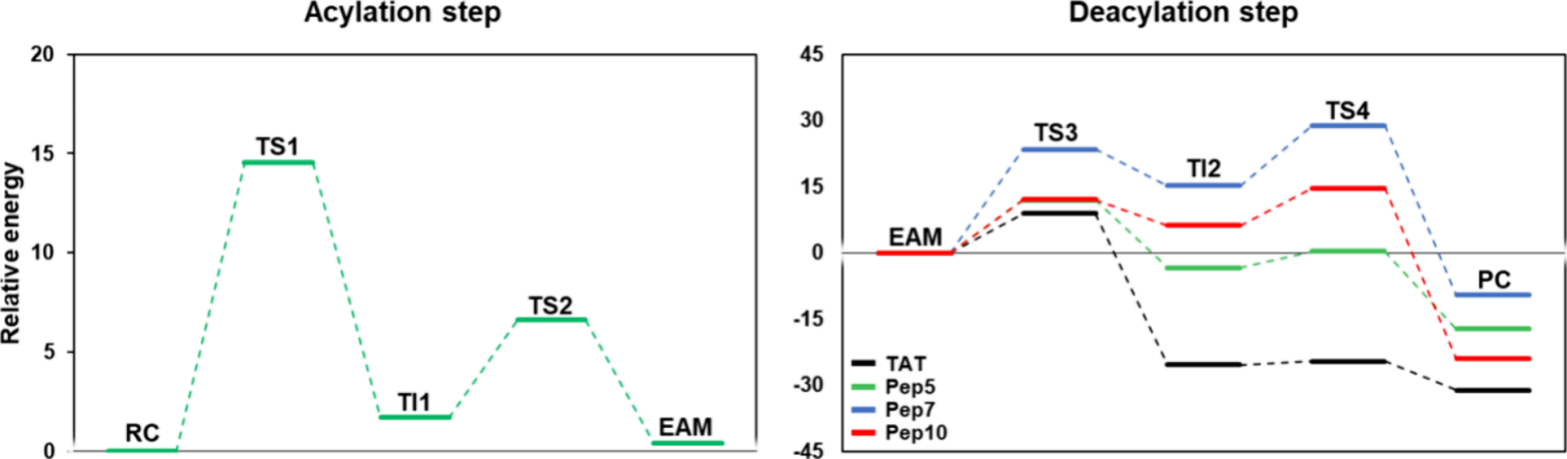
Free energy profiles for PNA-peptide conjugates derived from QM/MM metadynamics calculations in water. The energetic values were calculated with B3LYP-GPW/MM and are given in kcal mol^–1^. Acylation: Δ*G*^‡^**TS1** 14.5; **TI1**: 1.7; **TS2**: 6.6; **EAM**: 0.4; deacylation: **TS3** (**TAT**: 8.9; **Pep5**: 11.9; **Pep7**: 23.5; **Pep10**: 12.1); **TI2** (**TAT**: −25.4; **Pep5**: −3.4; **Pep7**: 15.2; **Pep10**: 6.2); **TS4** (**TAT**: −24.7; **Pep5**: 0.3; **Pep7**: 28.9; **Pep10**: 14.6); **PC** (**TAT**: −31.2; **Pep5**: −17.3; **Pep7**: −9.6; **Pep10**: −24.0).

**Figure 4:**
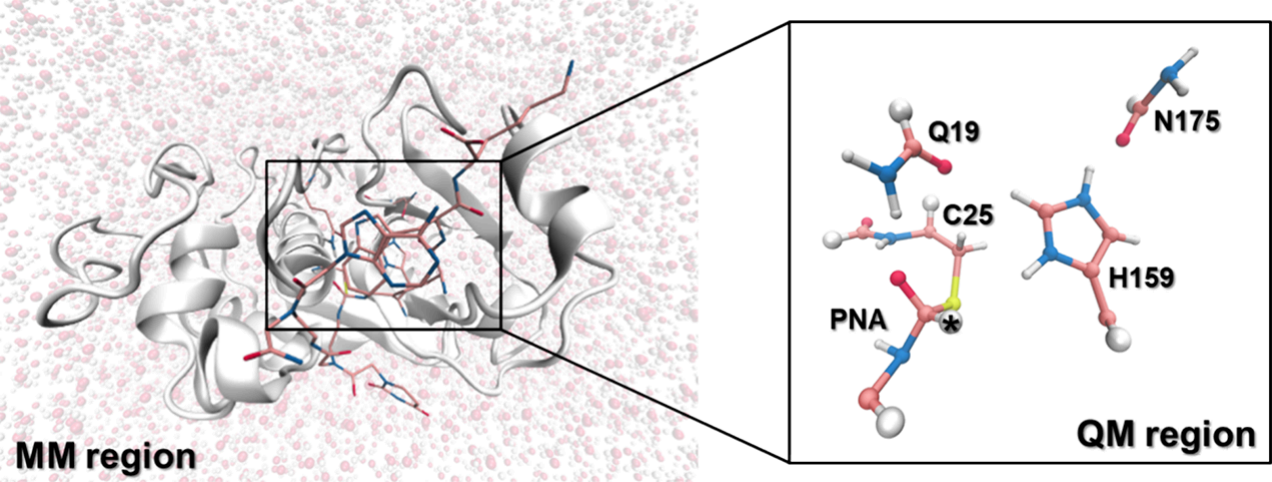
Representative structure of a system under study (PNA-Pep10). On the left, the depicted substrate is the PNA-Pep10 system treated with MM. At the right, the zoomed-in structure represents the QM region of the model. In white balls, the hydrogen atoms added to the interface of the QM and MM regions (link-atom approach). The atom with the asterisk represents the first amino acid of the peptide or the water or alcohol moieties of each reactional phase.

For the conjugation of the synthetic nucleic acid to the delivery peptides, first, the PNA must attach to the enzyme pocket similar to BCME. Both structures present resemblances such as the elongated backbone and the presence of an aromatic ring, which could display similar interactions in the active site (SI Figure S1). After observing BCME binding to the enzyme, we modeled the first two nucleobases (adenine and uracil) of the PNA molecule that could directly interact with the active site residues, along with the first three amino acids from the peptides. The free energy profiles were obtained from QM/MM metadynamics simulations (Figure 3). Atoms treated with QM and MM are depicted in Figure 4.

#### 3.2.1 Acylation

**Figure 5:**
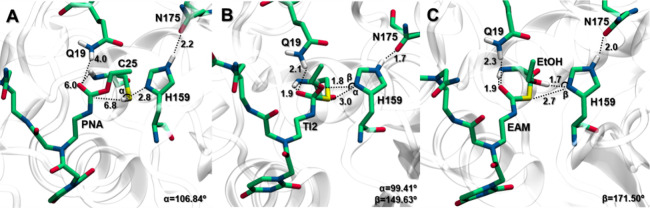
Active site pocket reference structures of the lowest-energy stationary points **RC**, **TI1**, and **EAM** (A–C, respectively) of a peptide nucleic acid (PNA). Key distances are given in Å, and free ethanol is shortened to EtOH.

The entry of a PNA molecule in the enzyme’s active site (Figure 5A) culminates in the release of ethanol to generate the **EAM**. Unlike BCME, the mechanism for PNA is concerted, although the interactions that they present are mostly similar. For the screening of the acylation stage, we relied on the use of collective variables CV_1_ and CV_2_ (Figure 2). The chosen coordinates for the reaction represent the deprotonation of C25 by H159 (d_1_) and the formation of an S–C bond upon nucleophilic attack of C25 to the PNA molecule (d_2_), leading to the formation of the tetrahedral intermediate **TI1** structure. After that, the coordinates corresponding to the cleavage of the PNA’s C–O bond (d_3_) and deprotonation of H159 by the ester oxygen of the PNA (d_4_) lead the reaction to the release of an ethanol molecule (**EAM**).

From the obtained energy values along the reaction coordinates, we found a free energy barrier Δ*G*^‡^ of 14.53 kcal mol^–1^ for the proton transfer from C25 to H159 and the nucleophilic attack on the PNA by C25 (**TS1**). This value is similar to that observed for the BCME’s control reaction (0.89 kcal mol^–1^ higher,^61^SI Figure S5). The formation of **TI1** corresponds to a local minimum of 1.69 kcal mol^–1^. In this structure, the newly formed S–C bond is at 1.80 Å, and the transferred proton (H_H159_^δ^) is 1.79 Å from the ester oxygen of the PNA at 149.63° and 2.96 Å from S_C25_ at 99.41°. Concerning the oxyanion hole residues, the PNA’s carbonyl oxygen presents hydrogen bonds with Q19 (2.14 Å) and C25’s amide group (1.86 Å) (Figure 5B). The reaction then moves forward through **TS2**, which has an energy barrier of 6.60 kcal mol^–1^, leading to the formation of **EAM** (Figure 5C) with the release of ethanol, 6.21 kcal mol^–1^ below **TS2**.

#### 3.2.2 Deacylation

**Figure 6:**
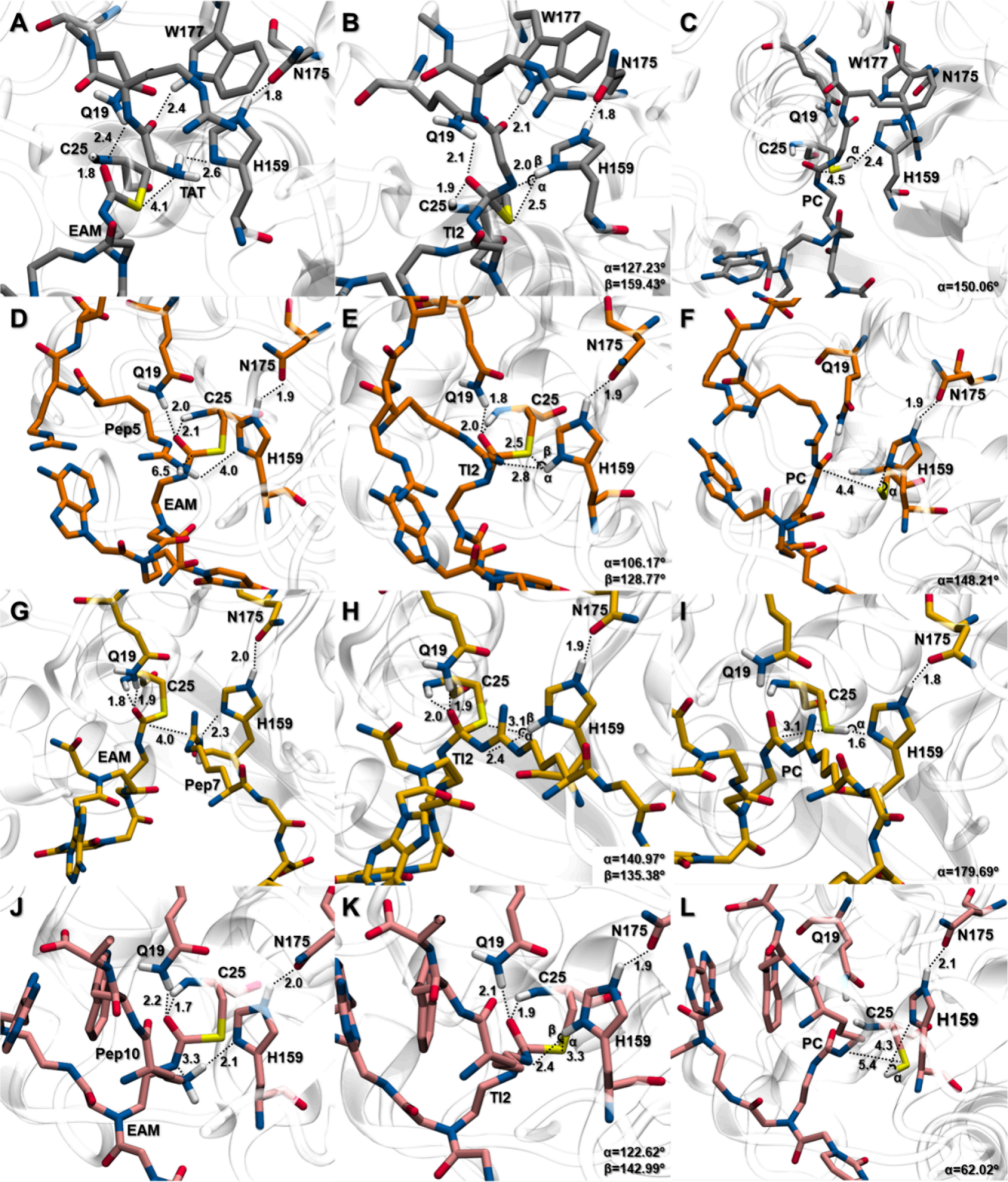
Active site pocket reference structures of the lowest-energy stationary points **EAM** (A, D, G, J), **TI2** (B, E, H, K), and **PC** (C, F, I, L) of a peptide nucleic acid (PNA) with a cell delivery peptide (by rows: TAT, Pep5, Pep7, and Pep10). For simplicity, only the first atoms of PNA and peptide are shown. Key distances are given in Å.

Contrary to BCME’s hydrolysis reaction where a water molecule performs a nucleophilic attack on the thioester, the aminolysis reaction on the PNA-CPP conjugates implies the nucleophilic attack to the thioester by a peptide followed by the release of the conjugated product. Here, we have performed simulation studies with the TAT CPP as well as the three best ranked delivery peptides Pep5, Pep7, and Pep10, designed in our previous work.^35^ In this step, the collective variables CV_1_ and CV_2_ represent the formation of the second TI (**TI2**, Figure 6B,E,H,K) with the deprotonation of the peptide by H159 (d_4_) and the nucleophilic attack of the peptide’s nitrogen atom to the PNA (d_3_, Figure 6A,D,G,J). The establishment of the **PC** structure (Figure 6C,F,I,L) is then obtained by cleavage of the S–C bond between C25 and the substrate (d_2_) and deprotonation of H159 by C25 (d_1_).

The PMF for this step is listed in Figure 3 (right). The free energy barrier for this reaction is related to two transition states: **TS3** for the TAT and Pep5 peptides (Δ*G*^‡^ of 8.91 and 11.93 kcal mol^–1^, respectively) while for peptides Pep7 and Pep10, it is **TS4** (Δ*G*^‡^ of 28.87 and 14.60 kcal mol^–1^, respectively). The formation of **TI2** corresponds to a local minimum of −25.36, −3.38, 15.18, and 6.18 kcal mol^–1^ for TAT, Pep5, Pep7, and Pep10, correspondingly. On average, in this structure (**TI2**, Figure 6B,E,H,K), the S–C bond is at 1.87 ± 0.05 Å, and the transferred H_H159_^δ^ proton is 2.52 ± 0.47 Å from the nitrogen atom of the substrate amide (128.77–159.43°) and 2.76 ± 0.39 Å from S_C25_ (106.17–140.97°). Concerning the oxyanion hole residues, the carbonyl oxygen of the substrate presents a 1.96 ± 0.14 Å hydrogen bond with Q19 and 1.96 ± 0.09 Å with C25’s amide group. The reaction then moves forward through **TS4**, leading to the formation of the **PC** structure (Figure 6C,F,I,L), which is 6.54, 17.62, 38.45, and 38.63 kcal mol^–1^ below that of **TS4** for TAT, Pep5, Pep7, and Pep10, respectively.

#### 3.2.3 Overall Reaction Mechanism

The results suggest that the rate-limiting step of the reaction is the barrier associated with **TS1** for three peptides (TAT, Pep5, and Pep10) with a Δ*G*^‡^ of 14.53 kcal mol^–1^, just 0.89 kcal mol^–1^ higher than the value calculated to BCME, and in close agreement with the experimental value of 15.7 kcal mol^–1^ for the BCME reaction. For the predicted enzymatic synthesis of the PNA-Pep7 conjugate, the rate-limiting step shifted to **TS4**, with a Δ*G*^‡^ of 28.87 kcal mol^–1^. This could be due to the different spatial positioning of the nucleophilic arginine compared with Pep5, which makes the peptide approach and interact with the R-domain of the enzyme rather than with the L-domain, and different from Pep10, which interacts with the PNA via π–π stacking. Even though different initial TI2 structures were modeled for Pep7 to resemble Pep5, the structure would change during MD simulations, and when positional restraints were imposed, metadynamics simulations did not find a minimum energy path to successful product formation. For the PNA-Pep10 system, the barriers associated with **TS1** and **TS4** are only slightly different (**TS4** is 0.07 kcal mol^–1^ higher than **TS1**). This could be due to an error associated with the method. Nevertheless, for all the systems under study, the free energy of **PC** is lower than that of **RC**, resulting in a negative free energy change, which points to a thermodynamically favorable reaction.

#### 3.2.4 Active Site Analysis

In the acylation step of BCME and PNA, although the rate-limiting step displays similar energies in TS1, the reaction is exergonic for BCME, with an energy of −28.4 kcal mol^–1^ on the acylation product (EAM) relative to that of RC, while for PNA, the reaction is predicted to be slightly endergonic or close to neutral (EAM is 0.4 kcal mol^–1^ higher than that of RC), which could affect conversion and be a bottleneck for deacylation. Additionally, in the deacylation stage of the Pep7 and Pep10 peptides, the energy associated with the forward reaction is higher than the reverse reaction, meaning that the forward reaction is slower. Thus, we wanted to scan the energy profiles of enzyme variants to verify if we could find a mutation that could (i) ameliorate the acylation step endergonic reaction issue; (ii) predict the synthesis of the PNA-Pep7 conjugate, by ameliorating the acceptance of the substrate into the active site, and (iii) improve the reaction rates of the deacylation step in both Pep7 and Pep10 systems, while also (iv) exploring the role of different residues in or near the active site. We started with the more complex peptide system (PNA-Pep10), and only advanced for PNA-Pep7 if the results from the latter were promising, as we wanted to find a variant that would work with these two substrates.

First, we tried to increase the stability of catalytic histidine H159, as its positioning and orientation are vital for the reaction to occur. By altering the N175 residue to an aspartic acid (N175D), we hypothesized that we could increase the number of interactions between them from one to two (H_H159_^ε^ to two oxygens of N175D), but it led to the distancing of H159 from S_C25_ up to 7.57 and 7.62 Å to the peptide nitrogen atom, after 50 ns of MD. Given this result, we kept the histidine-stabilizing N175 and tried to increase the stability of H159 by elongating V161, localized on the back between these two residues. We hypothesized that a longer side chain would allow the residue to also form interactions with H159, further stabilizing it. However, V161E’s TI2 structure changed the conformational space in such a way that H159 and N175 no longer interacted, and the helix where Q19 is located changed its conformation, with the peptide taking its space near the L-domain side. Thus, we shortened the long side chain of the glutamic acid by replacing the valine residue with an aspartic acid (V161D). The TI2 structure exhibited the H_H159_^ε^ atom interacting with the oxygen of V161D (1.79 Å) but now interacting with the nitrogen atom of the N175’s amino group (2.53 Å) rather than with the oxygen. The distance of H_H159_^δ^ to S_C25_ and the nitrogen atom of the peptide is 2.73 and 2.23 Å, respectively. Nevertheless, we still observed a push of the peptide to the L-domain side, repositioning the W177. As the literature reports this residue to be important for the initiation of the reaction by capping the His-Cys ion-pair,^64^ we did not proceed to the PMF scan.

Following the latter and as we observed that the W177 residue was sometimes moved by the motions of the peptide during MD simulations, we wanted to evaluate if we could find a suitable TI2 structure in which this residue was substituted with another one. First, we reduced the space occupied by the W177 side chain by mutating it with tyrosine (W177Y), hypothesizing that it could form hydrogen bonds with the asparagine while maintaining the polar and aromatic character of this residue. However, the system behavior was similar to that observed in the V161D mutation: the tyrosine’s aromatic ring points outside of the enzyme, not performing the putative cap function of the tryptophan, and the peptide moved to the L-domain side. Thus, we also did not proceed to scan the reaction energies.

**Figure 7:**
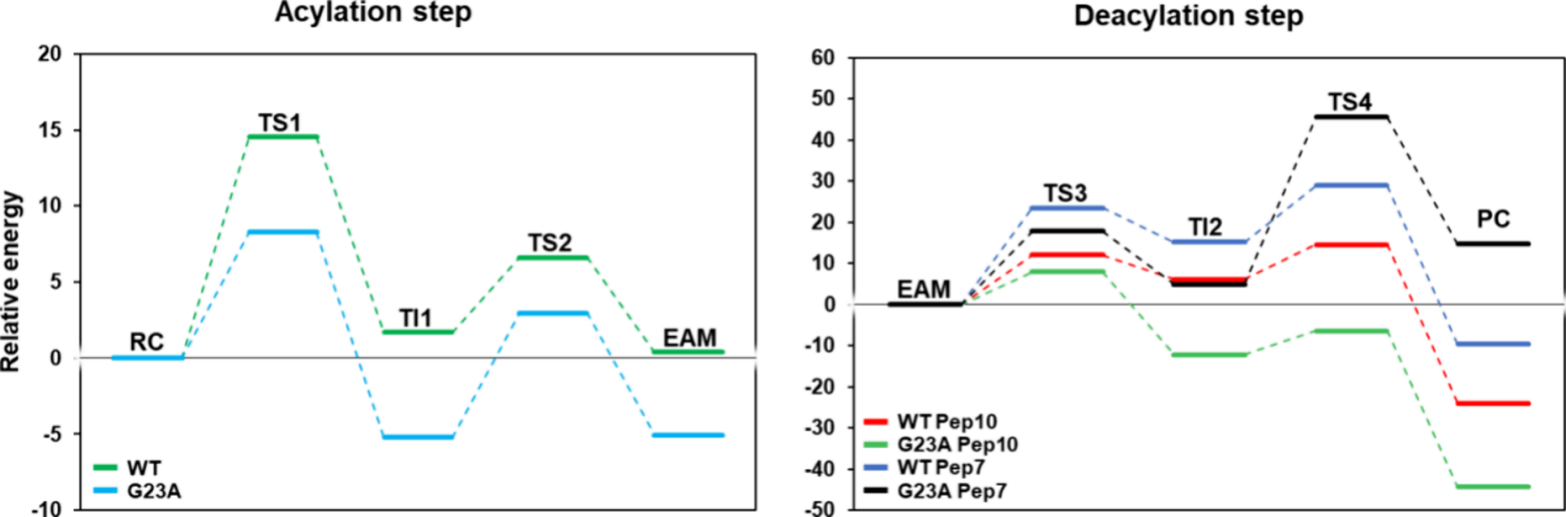
Free energy profiles for PNA-peptide conjugate enzymatic synthesis with the papain G23A variant derived from the QM/MM metadynamics calculations in water. The energetic values were calculated with B3LYP-GPW/MM and are given in kcal mol^–1^. Acylation: **TS1** (**WT**: 14.3; **G23A**: 8.29); **TI1** (**WT**: 1.7; **G23A**: −5.21); **TS2** (**WT**: 6.6; **G23A**: 2.97); **EAM** (**WT**: 0.4; **G23A**: −5.07); deacylation: **TS3** [**Pep7** (**WT**: 23.5; **G23A**: 17.83); **Pep10** (**WT**: 12.1; **G23A**: 7.99)]; **TI2** [**Pep7** (**WT**: 15.2; **G23A**: 4.91); **Pep10** (**WT**: 6.2; **G23A**: −12.16)]; **TS4** [**Pep7** (**WT**: 28.9; **G23A**: 45.57); **Pep10** (**WT**: 14.6; **G23A**: −6.40)]; **PC** [**Pep7** (WT: 9.6; **G23A**: 14.74); **Pep10** (**WT**: −24.0; **G23A**: −44.31)].

To enhance the substrate’s stability in the pocket, we tried to increase the number of interactions with the surrounding residues, by mutating the glycine G23 residue. Following our previous approach, we modified this residue to amino acids with longer side chains to approximate it to the substrate. First, we performed the G23N mutation, but after 50 ns of MD simulation, the new residue did not present any interaction with the substrate, with the side chain pointing outside of the protein and moving the L-domain. Thus, we decreased the side chain length by replacing this residue with a threonine (G23T), but the achievement of a TI2 structure was not possible, as the overall conformation of the V-cleft changed and no residue that took part in the reaction was near the substrate or active site (Q19, H159, and N175). At last, we decided to just add a methyl group to the glycine residue (G23A) and, even though the structure was very similar to the WT enzyme and we did not observe an increase in the number of interactions with the substrate, the overall conformation led us to proceed with the scan of the reaction mechanism with QM/MM metadynamics (Figure 7).

For the acylation step, comprising the entry of the PNA into the active site, we observed a decrease in the energy barrier associated with **TS1** of about 6.25 kcal mol^–1^ (Δ*G*^‡^: 8.29 kcal mol^–1^) compared with the WT papain. The reaction moves forward toward the **EAM** structure through **TS2** with an energy of 2.97 kcal mol^–1^, which is 8.19 kcal mol^–1^ higher than that of **TI1**. The G23A variant seems to perform energetically better to the acceptance of the nucleic acid in the active site compared to the native enzyme. Regarding the peptide entrance and covalent binding to the PNA, Pep7’s **TS4** rate-limiting step failed significantly compared to the WT, with a Δ*G*^‡^ of 45.57 kcal mol^–1^, 16 kcal mol^–1^ higher than the native enzyme. For this system, the free energy of **PC** is higher than that of **RC**, resulting in a positive free energy change, culminating in a nonspontaneous and thermodynamically unfavorable reaction. However, for Pep10, we can observe a difference between **TS3** and **TS4**, culminating in an improvement in the energetic profile with an energy decrease in **TS3** (Δ*G*: 7.99 kcal mol^–1^) and **TS4** (Δ*G*: −6.40 kcal mol^–1^). The PNA-Pep10 G23A variant reaction now clearly displays **TS1** as the rate-limiting step, with a lower Δ*G*^‡^ of 8.29 kcal mol^–1^, and the forward reaction’s free energy barrier at the deacylation step is now lower than the reverse (5.75 versus 20.14 kcal mol^–1^), which points to a higher reaction rate. Although we could not find a papain variant that might potentially synthesize the PNA-CPP conjugate for both Pep7 and Pep10 peptides, the G23A variant significantly improved the reaction energies from the PNA-Pep10 system and points to a successful enzymatic synthesis with lower associated energies and higher reaction rates.

### 3.3 Peptide Nucleic Acid-Delivery Peptide Conjugation in Organic Solvent

In the previous section, we explored the feasibility of the papain enzyme to conjugate the PNA to delivery peptides via the formation of peptide bonds on water solvent, through the PNA’s ester terminal and the peptides’ amide terminal. However, we also wanted to explore the capacity of the papain enzyme to perform the reaction via the carboxylic acid of the peptide and the PNA’s terminal amide. We selected the peptides with positive internalization results from our previous work (TAT and Pep5)^35^ for this screening, modeling the first six residues of each peptide (TAT: RRRPQC and Pep5: KRFGGAC) and the first two nucleobases of PNA (cytosine and adenine).

**Figure 8:**
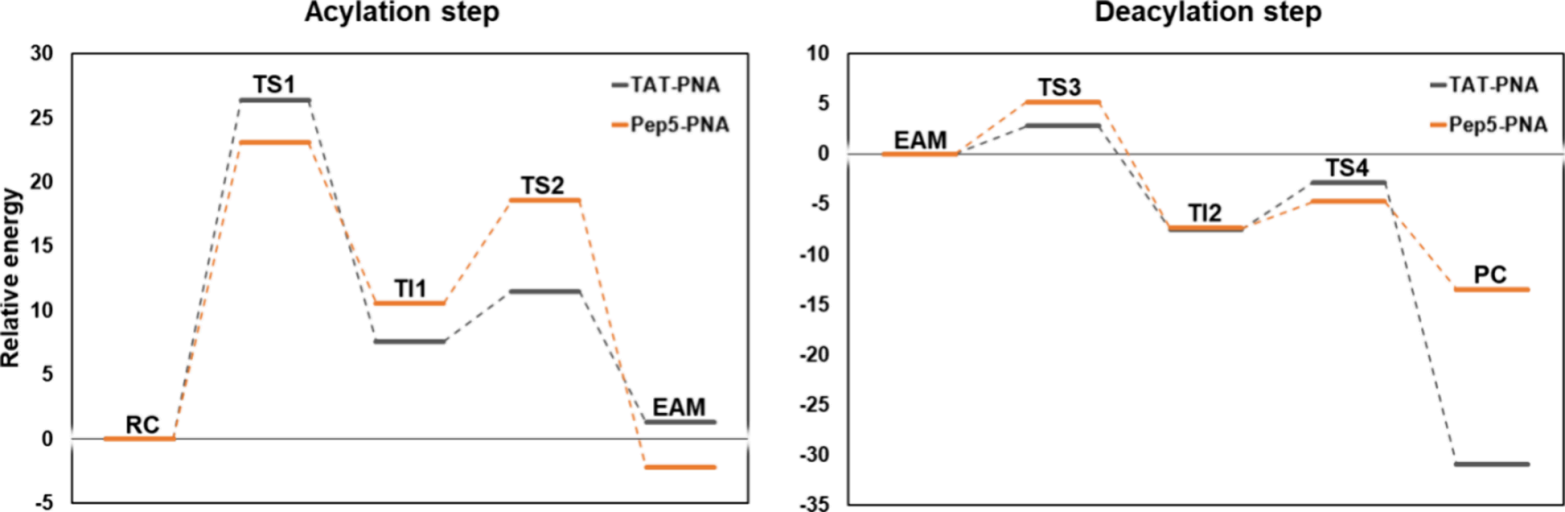
Free energy profiles for PNA-CPP_TAT_ and PNA-CPP_Pep5_ conjugate reaction derived from the QM/MM metadynamics calculations in an organic solvent (20% methanol). The energetic values were calculated with B3LYP-GPW/MM and are given in kcal mol^–1^. Δ*G*^‡^**TS1** (**TAT-PNA**: 26.3; **Pep5-PNA**: 23.1); **TI1** (**TAT-PNA**: 7.6; **Pep5-PNA**: 10.6); **TS2** (**TAT-PNA**: 11.5; **Pep5-PNA**: 18.5); **EAM** (**TAT-PNA**: 1.29; **Pep5-PNA**: −2.2); **TS3** (**TAT-PNA**: 2.8; **Pep5-PNA**: 5.2); **TI2** (**TAT-PNA**: −7.6; **Pep5-PNA**: −7.4); **TS4** (**TAT-PNA**: −2.9; **Pep5-PNA**: −4.8); **PC** (**TAT-PNA**: −30.9; **Pep5-PNA**: −13.5).

After literature research regarding the best organic solvents for this reaction, we found that a solvent containing 20% methanol was reported to provide a good yield in polypeptide synthesis.^15^ Additionally, peptides can be acquired with an additional C-terminal cysteine residue to allow the attachment of a fluorophore label; thus, in these simulations, this amino acid is the one attacking the catalytic C25 in both peptides. Accordingly, while for the reaction in water the acylation step corresponded to the PNA entry and the deacylation to the peptide attachment, now the peptide will bond to the enzyme during the acylation step followed by the nucleophilic attack of the PNA during deacylation and subsequent conjugation to the peptide. The PMF for these reactions is presented in Figure 8.

#### 3.3.1 TAT-PNA

**Figure 9:**
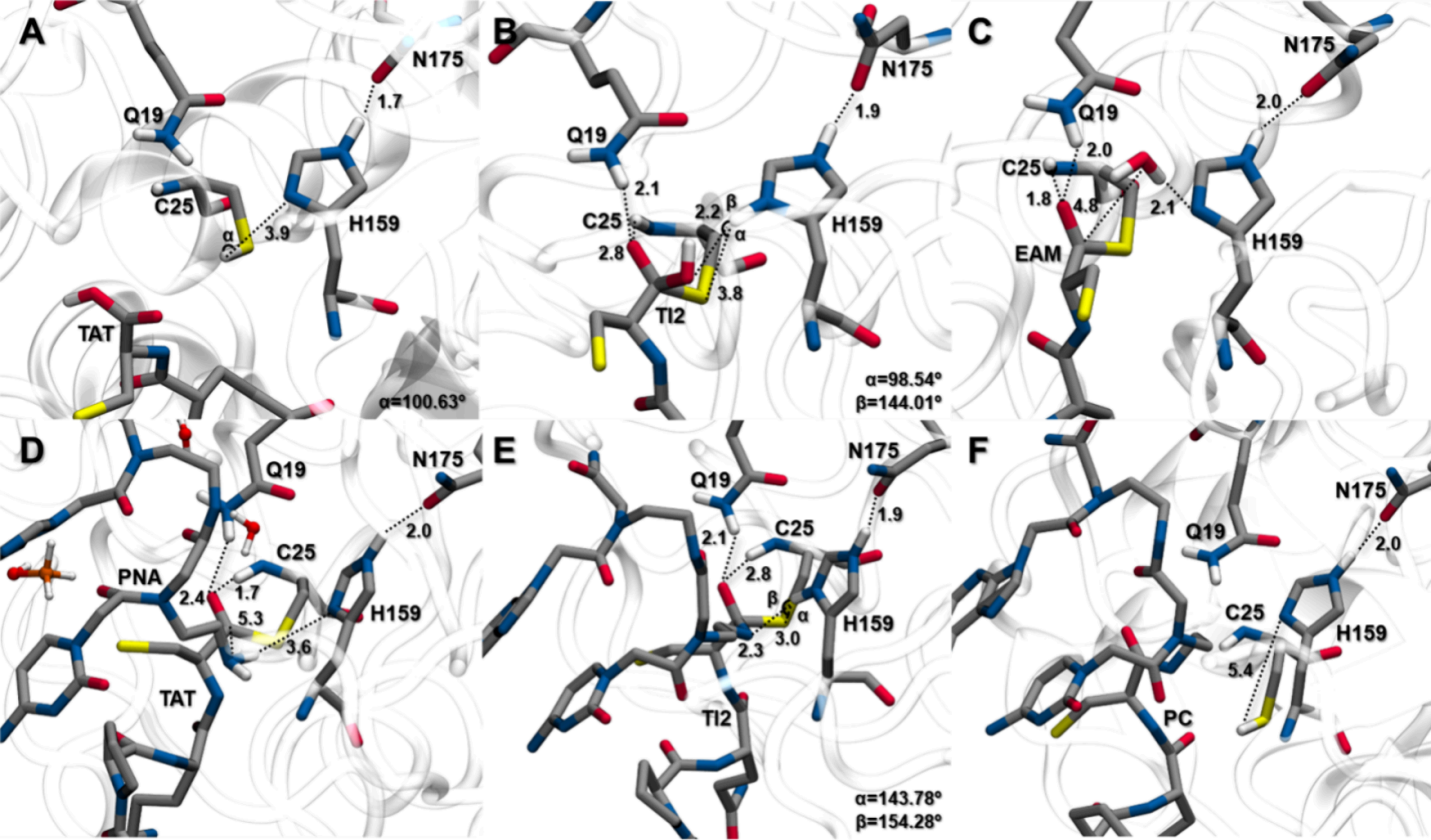
Active site pocket reference structures of the lowest-energy stationary points **RC**, **TI1**, **EAM** (acylation), **EAM** (deacylation), **TI2**, and **PC** (A–F, respectively) of a peptide nucleic acid (PNA) with a cell delivery peptide (TAT). In panel (D), the solvent (water and methanol) within 5 Å of the substrate is shown as stick-and-ball drawing. For simplicity, only the first atoms of the PNA and peptide are shown. Key distances are given in Å, and PC refers to conjugated product PNA-CPP_TAT_.

The entry of the TAT peptide in the active site culminates in the release of a water molecule to generate the **EAM**. For the screening of the acylation stage, the chosen CV coordinates represent the deprotonation of C25 by H159 (d_1_) and the formation of an S–C bond upon nucleophilic attack of C25 to the substrate (d_2_), leading to the formation of the **TI1** structure. After that, the coordinates corresponding to the cleavage of the TAT’s C–O bond (d_3_) and deprotonation of H159 by the hydroxyl oxygen of the peptide (d_4_) lead the reaction to the release of a water molecule (**EAM**). The PMF for this step is listed in Figure 8 (left). From the obtained energy values along the reaction coordinates, we found a free energy barrier Δ*G*^‡^ of 26.34 kcal mol^–1^ for the proton transfer from C25 to H159 and the nucleophilic attack on the substrate by C25 (**TS1**). The formation of **TI1** corresponds to a local minimum of 7.61 kcal mol^–1^. In this structure, the newly formed S–C bond is at 1.87 Å, and the transferred proton (H_H159_^δ^) is 2.18 Å from the hydroxyl oxygen of the peptide at 144.01° and 3.80 Å from SC25 at 98.54°. Concerning the oxyanion hole residues, the peptide’s carbonyl oxygen presents hydrogen bonds with Q19 (2.10 Å) and C25’s amide group (2.75 Å) (Figure 9B). The reaction then moves forward through **TS2**, which has an energy barrier of 11.49 kcal mol^–1^, leading to the formation of **EAM** (Figure 9C) with the release of water, 10.2 kcal mol^–1^ below **TS2**.

Regarding the deacylation phase, the PNA enters the active site (Figure 9D) and performs a nucleophilic attack on the thioester followed by the formation of the conjugated product. In this step, the collective variables CV_1_ and CV_2_ represent the formation of **TI2** with the deprotonation of the PNA by H159 (d_4_) and the nucleophilic attack of the PNA’s nitrogen atom to the peptide substrate (d_3_). The establishment of the **PC** structure is then obtained by the cleavage of the S–C bond between C25 and the substrate (d_2_) and deprotonation of H159 by C25 (d_1_). The PMF for this step is presented in Figure 8 (right). The free energy barrier for this reaction is related to **TS3**, with a Δ*G*^‡^ of 2.79 kcal mol^–1^. The formation of **TI2** corresponds to a local minimum of −7.56 kcal mol^–1^. In this structure (Figure 9E), the S–C bond is at 1.89 Å, and the transferred H_H159_^δ^ proton is 2.27 Å from the nitrogen atom of the substrate amide (154.28°) and 3.00 Å from SC25 (143.78°). Concerning the oxyanion hole residues, the carbonyl oxygen of the substrate presents a 2.11 Å hydrogen bond with Q19 and 2.76 Å with C25’s amide group. The reaction then moves forward through **TS4**, leading to the formation of the **PC** structure (Figure 9F), which is 28.13 kcal mol^–1^ below that of **TS4**.

#### 3.3.2 Pep5-PNA

**Figure 10:**
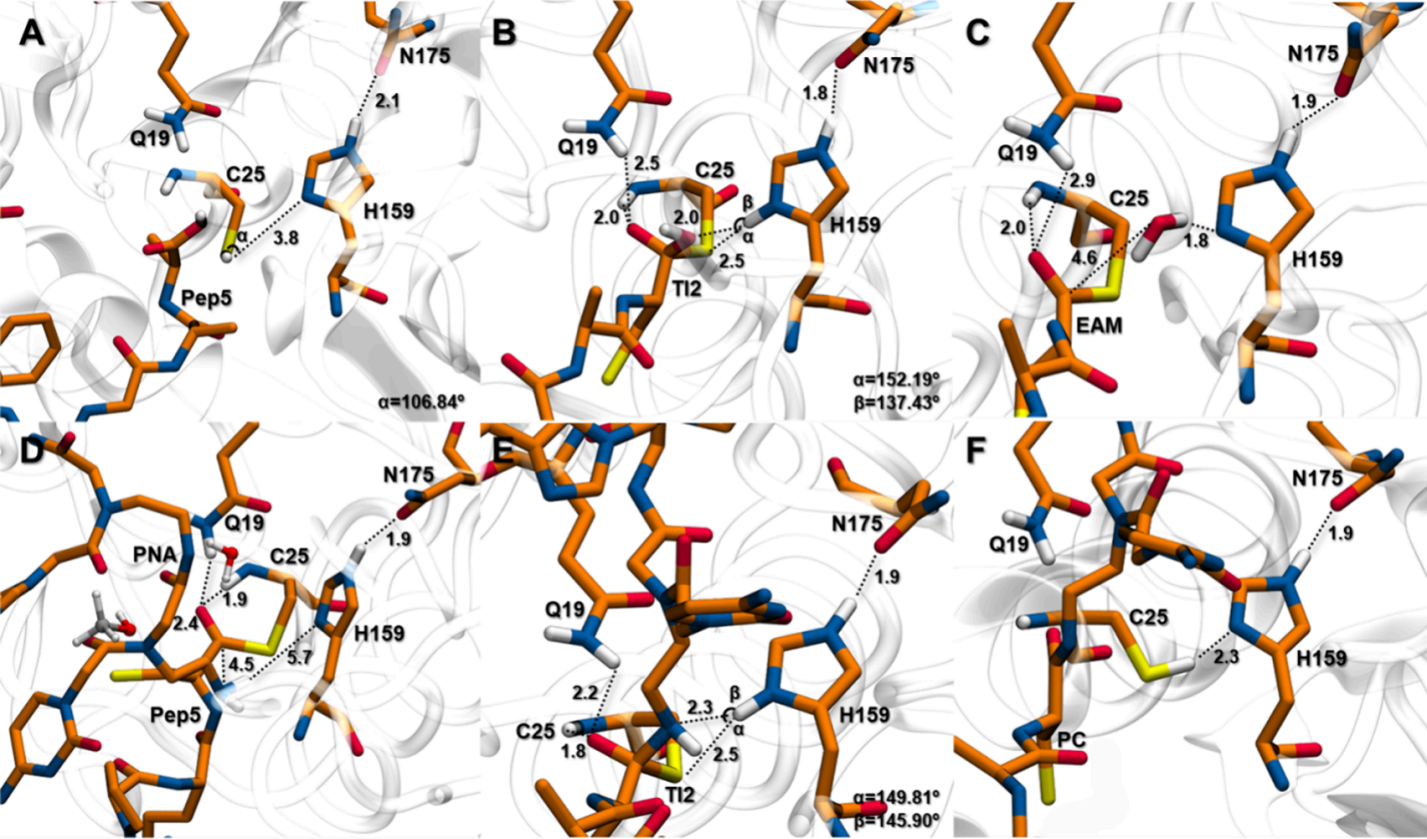
Active site pocket reference structures of the lowest-energy stationary points **RC**, **TI1**, **EAM** (acylation), **EAM** (deacylation), **TI2**, and **PC** (A–F, respectively) of a peptide nucleic acid (PNA) with a cell delivery peptide (Pep5). In panel (D), solvent (water and methanol) within 5 Å of the substrate is shown as stick-and-ball drawing. For simplicity, only the first atoms of the PNA and peptide are shown. Key distances are given in Å, and PC refers to the conjugated product PNA-CPP_Pep5_.

Similar to the previous reaction, the EAM is generated after entry of the Pep5 peptide in the active site (Figure 10A), leading to the release of a water molecule. For the screening of this reaction, the CVs are the same as for the TAT peptide. For the acylation phase, the PMF is shown in Figure 8 (left). We found a free energy barrier of Δ*G*^‡^ of 23.05 kcal mol^–1^ for the proton transfer from C25 to H159 and nucleophilic attack on the substrate by C25 (**TS1**). The formation of **TI1** corresponds to a local minimum of 10.56 kcal mol^–1^. In this structure, the formed S–C bond is at 1.76 Å, and the transferred proton (H_H159_^δ^) is 1.99 Å from the hydroxyl oxygen of the peptide at 1.7.43° and 2.48 Å from S_C25_ at 152.19°. Concerning the oxyanion hole residues, the peptide’s carbonyl oxygen presents a weak hydrogen bond with Q19 (2.48 Å) and C25’s amide group (1.97 Å) (Figure 10B). The reaction then moves forward through **TS2**, which has an energy barrier of 18.54 kcal mol^–1^, leading to the formation of **EAM** (Figure 10C) with the release of water, at −2.22 kcal mol^–1^.

Then, the deacylation step, equal to the previous reaction, concerned the PNA performing a nucleophilic attack on the thioester followed by the formation of the conjugated product. The PMF for this step is presented in Figure 8 (right). As observed with the TAT peptide, the free energy barrier for the deacylation reaction is also **TS3**, with Δ*G*^*‡*^ = 5.19 kcal mol^–1^. The formation of **TI2** corresponds to a local minimum of −7.40 kcal mol^–1^, similar to the previous reaction. In this structure (Figure 10E), the S–C bond is also at 1.89 Å, and the transferred H_H159_^δ^ proton is 2.25 Å from the nitrogen atom of the substrate amide (145.90°) and 2.54 Å from S_C25_ (149.81°). Concerning the oxyanion hole residues, the carbonyl oxygen of the substrate presents a 2.17 Å hydrogen bond with Q19 and 1.84 Å with C25’s amide group. The reaction then moves forward through **TS4**, leading to the formation of the PC structure (Figure 10F), which is 8.75 kcal mol_^–1^_ below that of **TS4**.

## 4 Conclusions

We have screened the capability of the papain enzyme to catalyze the synthesis of nucleic acid-cell delivery peptide conjugates. We started with the establishment of the initial structures via covalent molecular docking and molecular dynamics followed by the exploration of the reaction mechanism through QM/MM metadynamic simulations from which the energetic profiles were retrieved.

As an initial screening, we explored the hydrolysis reaction of α-*N*-benzoyl-L-citrulline methyl ester by the papain enzyme and assessed all of the associated barriers. Then, we studied the more complex PNA-CPP systems. Ligation of a PNA to a delivery peptide may enhance PNA’s stability and water solubility as well as its capacity to reach cells by crossing the cell membranes. Conjugation via chemoenzymatic pathways rather than SPPS could help overcome the drawbacks of the latter for the synthesis of large peptides and avoid the need for previous chemical modification on the molecules one wants to ligate.

Overall, TS1 was identified as the rate-limiting step in the reaction mechanism for both BCME and the PNA-CPP systems with Δ*G*^‡^ values of 13.64 and 14.53 kcal mol^–1^, respectively. However, for PNA-Pep7, TS4 is the rate-limiting step with a Δ*G*^‡^ of 28.87 kcal mol^–1^, while for PNA-Pep10, the energy barriers of TS1 and TS4 are similar, differing by only 0.07 kcal mol^–1^, which is within the method’s error. Additionally, while the acylation step is exergonic for BCME, it is slightly endergonic for PNA (0.4 kcal mol^–1^), which could be an obstacle to a unidirectional reaction.

Trying to predict a feasible reaction from reactants to products for PNA-Pep7 and trying to improve the overall reaction rates in this and the PNA-Pep10 systems, we proceeded to analyze the role of the active site residues of the papain enzyme by performing several mutations and running MD simulations to verify if TI structures were detected before scanning reaction energy pathways. From the several variants analyzed *in silico*, G23A improved the energies associated with the enzymatic conjugation of the PNA-Pep10 system, decreasing the energy of the acylation’s limiting step, with a TS1 Δ*G*^‡^ of 8.29 kcal mol^–1^, and stabilizing the EAM product by decreasing its energy in 5.47 kcal mol^–1^ compared to the WT papain, turning the slightly endergonic acylation reaction into exergonic.

Based on this screening, we might hypothesize that papain could easily accept other nucleic acid derivatives, as long as the first nucleobase rests in a position closer to that observed on the BCME and PNA’s aromatic ring (although the different nucleic acid derivatives’ backbones would induce different interactions with the residues along the active site). Also, not only the first amino acid of the peptide sequence is important for the kinetics of the reaction but also the remaining residues. Even though we just modeled the first three amino acids of each peptide, it is clear that the positioning in the active site is different, even with the same first amino acid (e.g., two arginines of Pep5 and an arginine followed by a glycine on Pep7). Additionally, localization toward the L-domain rather than the R-domain could have led to the 11.56 kcal mol^–1^ increase in Pep7’s **TS3** compared to Pep5 (which displayed similar energy to that of Pep10, which has a lysine as the first residue). The CPP TAT was the one with the lowest **TS3** associated energy (8.91 kcal mol^–1^), exhibiting the reverse sequence of Pep7 (GR rather than RG), highlighting the importance of considering the enzyme pockets and amino acid preferences for each subpocket.

Moreover, we also scanned the reaction in a 20% methanol organic solvent. This reaction implied the inversion of the order related to substrate entry, with the peptide performing the first nucleophilic attack and covalently binding to the enzyme’s cysteine residue followed by the PNA. For both TAT-PNA and Pep5-PNA systems, the scanned reactions suggest the viability of the conjugate synthesis with the WT enzyme, but the reaction rate would be slower, as expected. The limiting step for both reactions is **TS1**, as in the reaction in water, but the activation energy increased for both systems (14.53 kcal mol^–1^ in water versus 23.05 and 26.34 kcal mol^–1^ for Pep5 and TAT, respectively). This could be due to the more stabilized structure of the reactions in water, where the aromatic ring of the first PNA nucleobase interacts with the enzyme’s Y67 via π–π stacking on the L-domain. For the reactions in organic solvent, TAT does not display any interaction with the L-domain, while the phenylalanine residue of Pep5 approaches the L-domain but is far from Y67 (10.76 Å); the longer distance of this residue to the active site (fifth residue) compared to the first nucleobase of the reaction in water may hinder a better interaction and stabilization.

Although exploratory, by expanding the synthetic routes for nucleic acid–based therapies, we are contributing to an easier and greener synthesis of therapies that could be used to target several health issues related to differential gene expression, ameliorating the delivery of those pharmaceutics. Our results suggest that the WT papain enzyme could be used for the enzymatic synthesis of the PNA-CPP systems with three of the four tested peptides (TAT, Pep5, and Pep10). For Pep7, however, the rate-limiting step is TS4, which would hinder successful conjugation; this could be reasoned with the spatial arrangement of Pep7’s arginine toward the R-domain as opposed to the L-domain (compared to Pep5), and this binding mode may influence reaction energetics. Exploration of the active site residues did not help to overcome this result for PNA-Pep7 but helped to improve the catalytic landscape for PNA-Pep10 with the G23A variant.

## Supporting Information Available

Schematic model of the PNA and BCME molecules (Figure S1); free energy landscape for the reactions in water with the native enzyme (Figure S2), G23A variant (Figure S3) and native enzyme in an organic solvent (Figure S4); free energy profiles for BCME hydrolysis reaction (Figure S5) and representative structure of the BCME acylation stage (Figure S6) and deacylation stage (Figure S7) (PDF)
