# Supplementary material for: Papain-Mediated Conjugation of Peptide Nucleic Acids to Delivery Peptides: A Density Functional Theory/Molecular Mechanics Metadynamics Study in Aqueous and Organic Solvent
Source: J Phys Chem B. 2024 Jul 25;128(31):7500–12. doi: 10.1021/acs.jpcb.4c02294 (PMC11317979; doi:10.1021/acs.jpcb.4c02294)
Supplement: Supplementary file 1 — jp4c02294_si_001.pdf [file jp4c02294_si_001.pdf]

Supporting Information

Papain-Mediated Conjugation of Peptide Nucleic Acids to Delivery Peptides: A DFT/MM Metadynamics Study in Aqueous and Organic Solvent

Ricardo D. González[a,b,c] *and Alexandra T. P. Carvalho*[a.b.d*]

[a] CNC – Center for Neuroscience and Cell Biology, University of Coimbra, Portugal
[b] CIBB – Center for Innovative Biomedicine and Biotechnology, University of Coimbra, Portugal
[c] University of Coimbra, Institute for Interdisciplinary Research, Doctoral Programme in Experimental Biology and Biomedicine (PDBEB), Portugal
[d] Almac Sciences, Department of Biocatalysis and Isotope Chemistry, Almac House, 20 Seagoe Industrial Estate, Craigavon BT63 5QD, Northern Ireland, United Kingdom

* alexandra.carvalho@almacgroup.com

Table of Contents

Figure S1. Schematic model of a PNA and BCME 3
Figure S2. FEL from the QM/MM metadynamics in water (WT enzyme)..............................4
Figure S3. FEL from the QM/MM metadynamics in water (G23A variant)............................5
Figure S4. FEL from the QM/MM metadynamics in organic solvent).....................................6
Figure S5. Free energy profiles for BCME hydrolysis reaction... 7
Figure S6. Representative structures of BCME acylation stage 8
Figure S7. Representative structures of BCME deacylation stage... 9

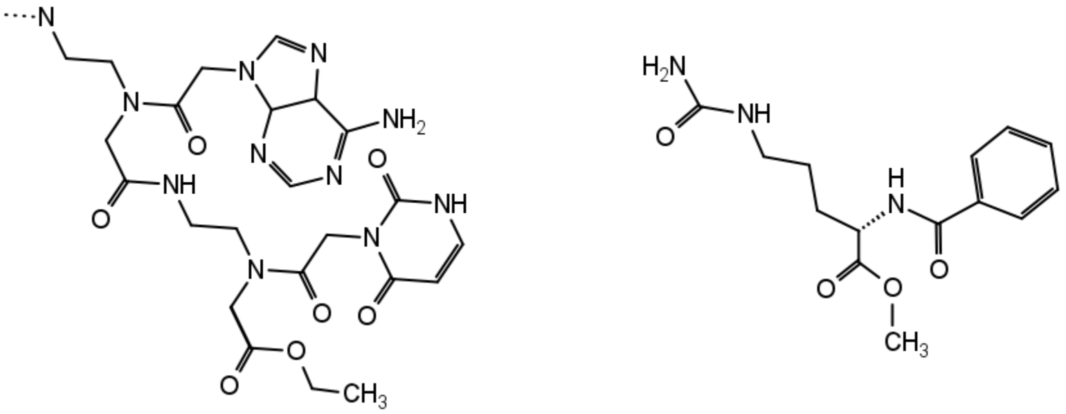

Figure S1. Schematic model of **left)** PNA with the first two nucleobases uracil and adenine, and **right)** α-*N*-benzoyl-L-citrulline methyl ester.

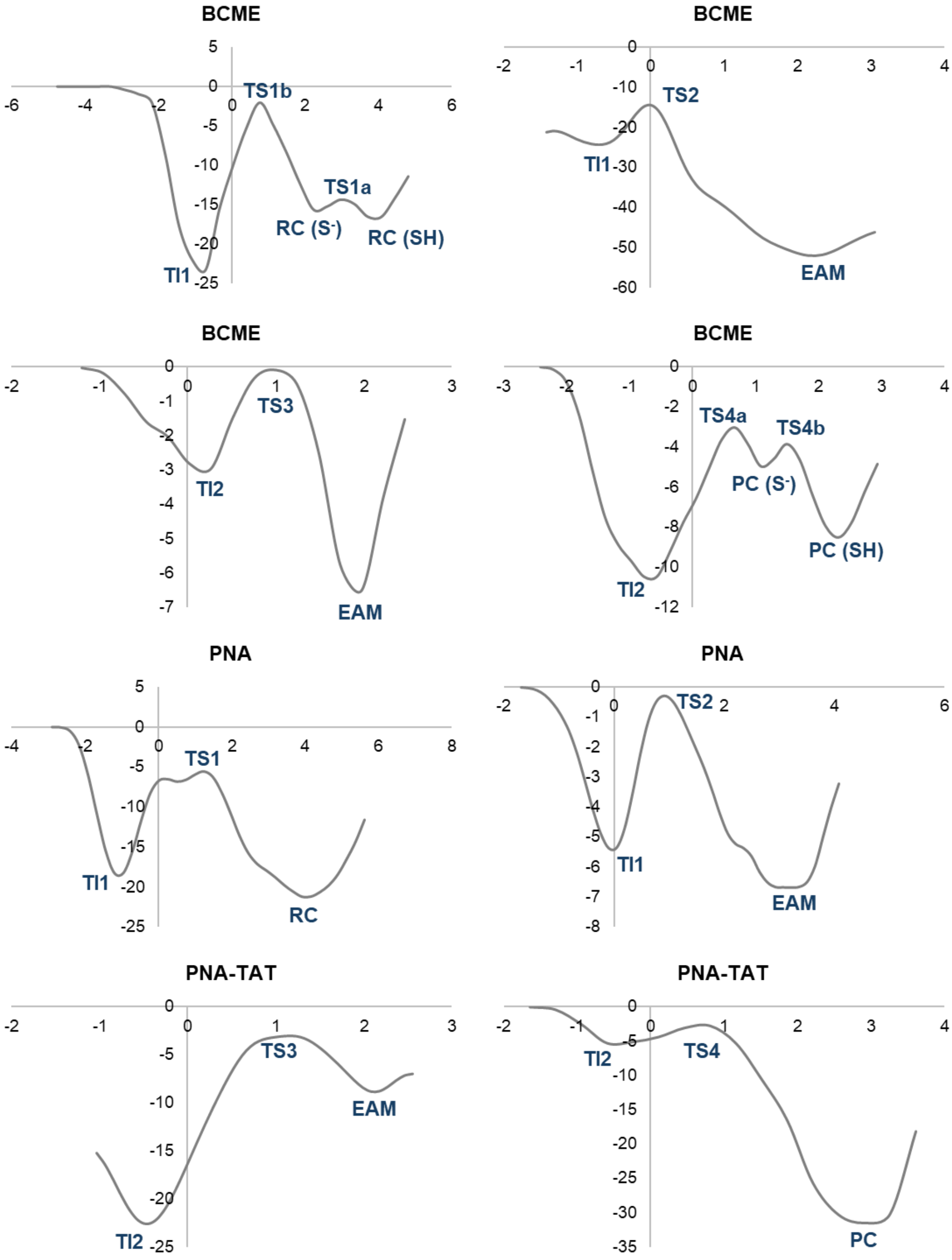

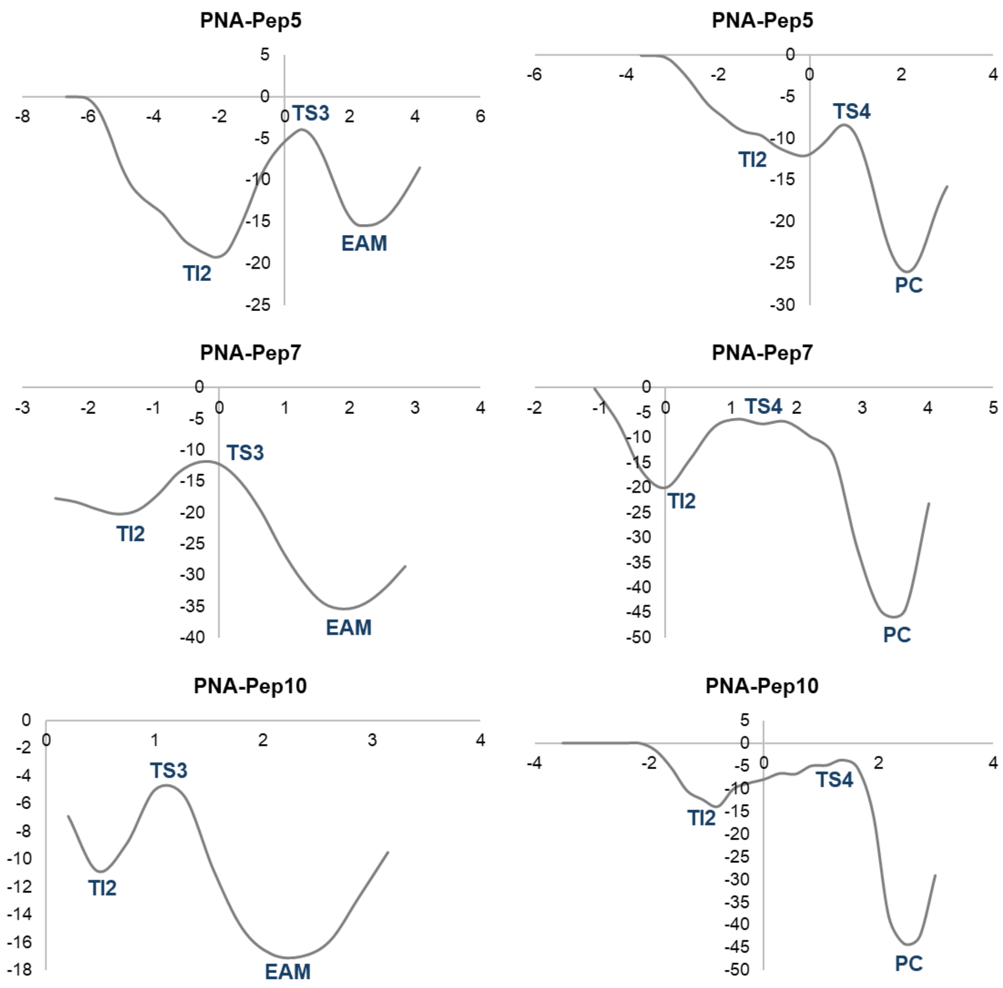

Figure S2. Free energy landscape from the QM/MM metadynamics in water, calculated with B3LYP-GPW/MM. In the vertical axis, the relative free energies are given in kcal mol^{-1}. In the horizontal axis, the position is given by the set of used collective variables. RC, reactant complex; PC, product complex; TI, tetrahedral intermediate; TS, transition state; EAM, enzyme-activated monomer.

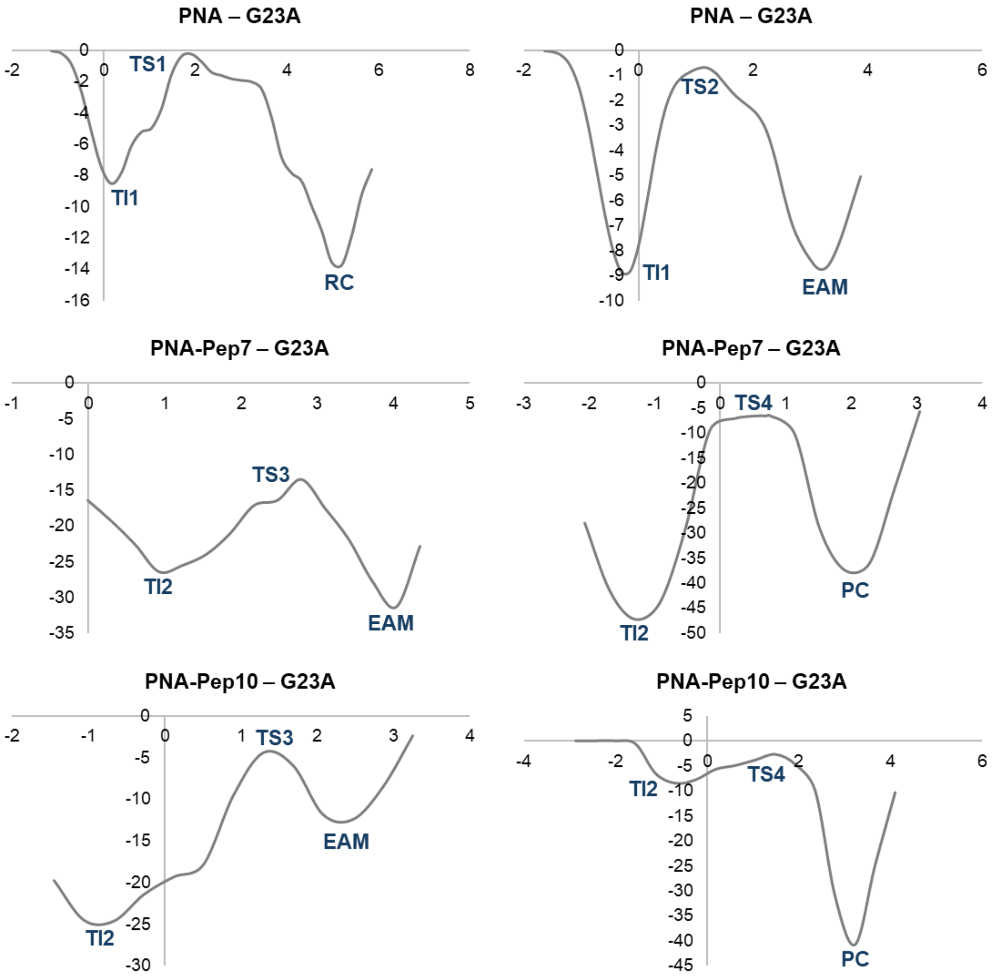

Figure S3. Free energy landscape from the QM/MM metadynamics in water, calculated with B3LYP-GPW/MM, regarding the enzyme variant G23A. In the vertical axis, the relative free energies are given in kcal mol^{-1}. In the horizontal axis, the position is given by the set of used collective variables. RC, reactant complex; PC, product complex; TI, tetrahedral intermediate; TS, transition state; EAM, enzyme-activated monomer.

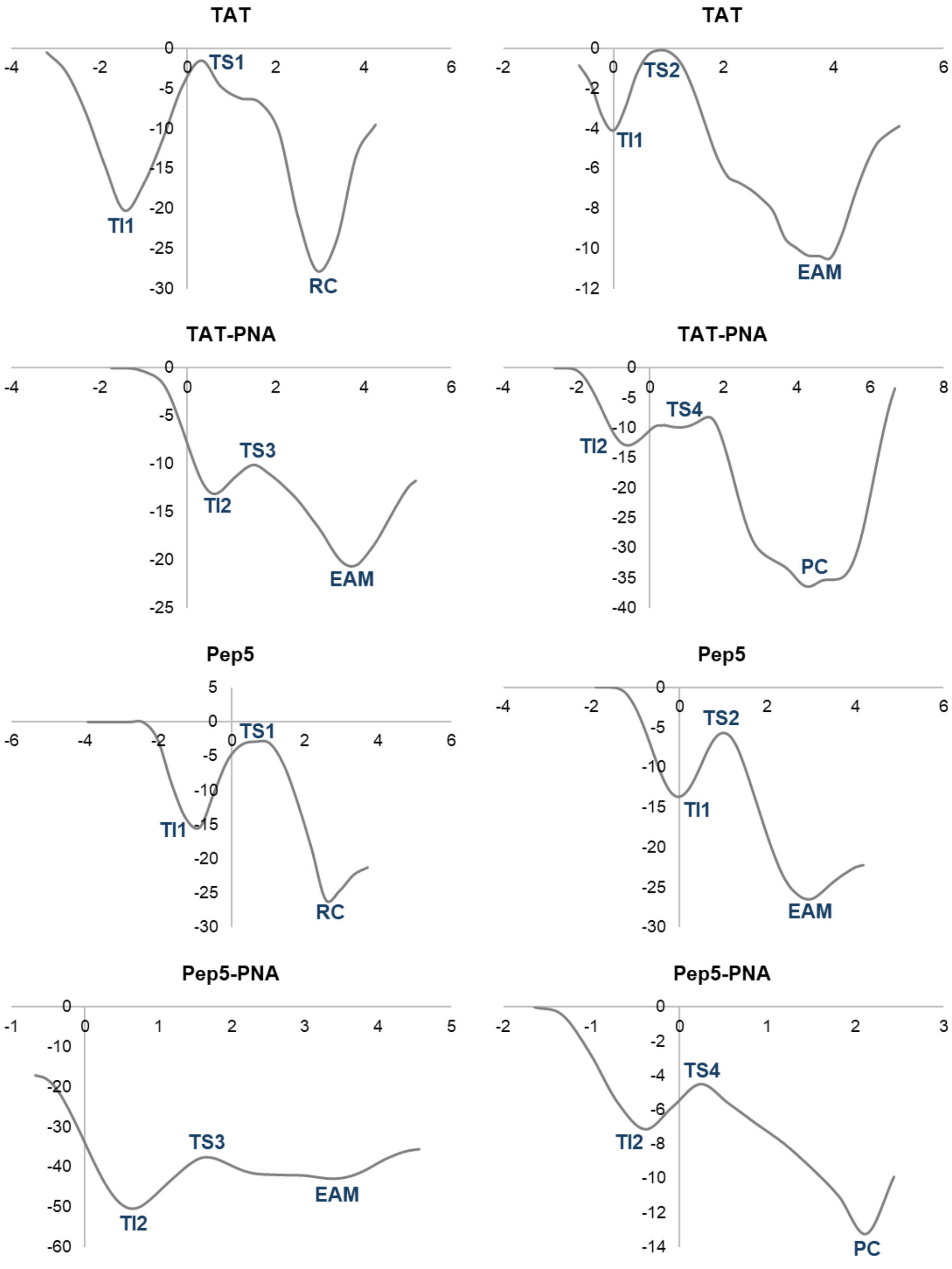

Figure S4. Free energy landscape from the QM/MM metadynamics in an organic solvent, calculated with B3LYP-GPW/MM. In the vertical axis, the relative free energies are given in kcal mol^{-1}. In the horizontal axis, the position is given by the set of used collective variables. RC, reactant complex; PC, product complex; TI, tetrahedral intermediate; TS, transition state; EAM, enzyme-activated monomer.

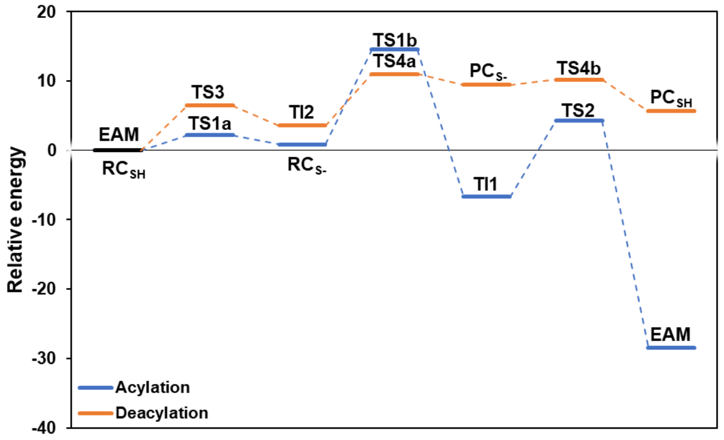

Figure S5. Free energy profiles for α-*N*-benzoyl-L-citrulline methyl ester derived from the QM/MM metadynamics calculations. The energetic values were calculated with B3LYP-GPW/MM and are given in kcal mol^{-1}: **TS1a**, 2.2; **RC**$_{S-}$, 0.9; **TS1b (Δ*G*‡)**, 14.5; **TI1**, -6.7; **TS2**, 4.3; **EAM**, -28.4; **TS3**, 6.4; **TI2**, 3.6; **TS4a**, 11.0; **PC**$_{S-}$, 9.5; **TS4b**, 10.2; **PC**$_{SH}$, 5.6.

Acylation. For BCME, the first part of the reaction is related to the release of methanol to generate the **EAM**. The reaction starts (**RC**$_{SH}$, Figure S6A) with the entry of the substrate into the active site pocket, following a stepwise mechanism. C25 presents van der Waals (vdW) interactions with A160 and H159, and the backbone oxygen presents hydrogen bonds with F28 and S29 amide groups (3.49 Å and 2.94 Å, respectively). H159 shows hydrogen bonds with L134's amide group (2.79 Å) and the amide oxygen of the stabilizing N175 (1.80 Å), vdW with A136 and π-π stacking with F141 and W177. The substrate only interacts with the oxyanion hole-forming Q19 via hydrogen bond between the negatively charged oxygen of BCME and the amide hydrogen of Q19 (1.93 Å) while entering the enzyme pocket. The first transition state **TS1a** has an energy barrier (Δ*G*) of 2.20 kcal mol^{-1} and leads to the transition of the thiol group to thiolate anion (**RC**$_{S-}$, Figure S6B). The thiol hydrogen is transferred to the Nδ of the H159, leading to changes in the C25 and Q19 interactions: the hydrogen bond between them shifts to a vdW; C25 forms vdW interactions with S176; and the amide group of Q19 starts interacting with the oxygen atom of C22 via hydrogen bonding (3.21 Å). **TS1b** presents a Δ*G*‡ of 13.64 kcal mol^{-1} and is resolved into the **TI1** structure (Figure S6C), which is -6.70 kcal mol^{-1}, with the nucleophilic attack by the C25 sulfur atom (S_{C25}) on the carbonyl carbon atom of BCME. The substrate now presents a covalent bond with C25 (1.86 Å) and the negatively charged oxygen atom has hydrogen bonds with the oxyanion hole residues (Q19: 2.02 Å, and the amide group of C25: 1.75 Å). The ester oxygen is close to $Nδ_{H159}$ (2.24 Å), pointing towards the forward reaction.

The reaction proceeds with the release of a methanol molecule to form the **EAM** structure (Figure S6D). Here, the positively charged H159 needs to transfer its proton to BCME's ester oxygen. This is achieved via **TS2**, which is 10.95 kcal mol^{-1} higher than **TI1** and 32.68 kcal mol^{-1} below **EAM**. The 10.28 kcal mol^{-1} difference between the energy required for **TI1** to transition forward (**TS2**) or reverse (**TS1**) is

corroborated by the smaller distance of the $H\delta_{H159}$ to the ester oxygen (2.24 Å) compared to the thiolate anion (4.14 Å).

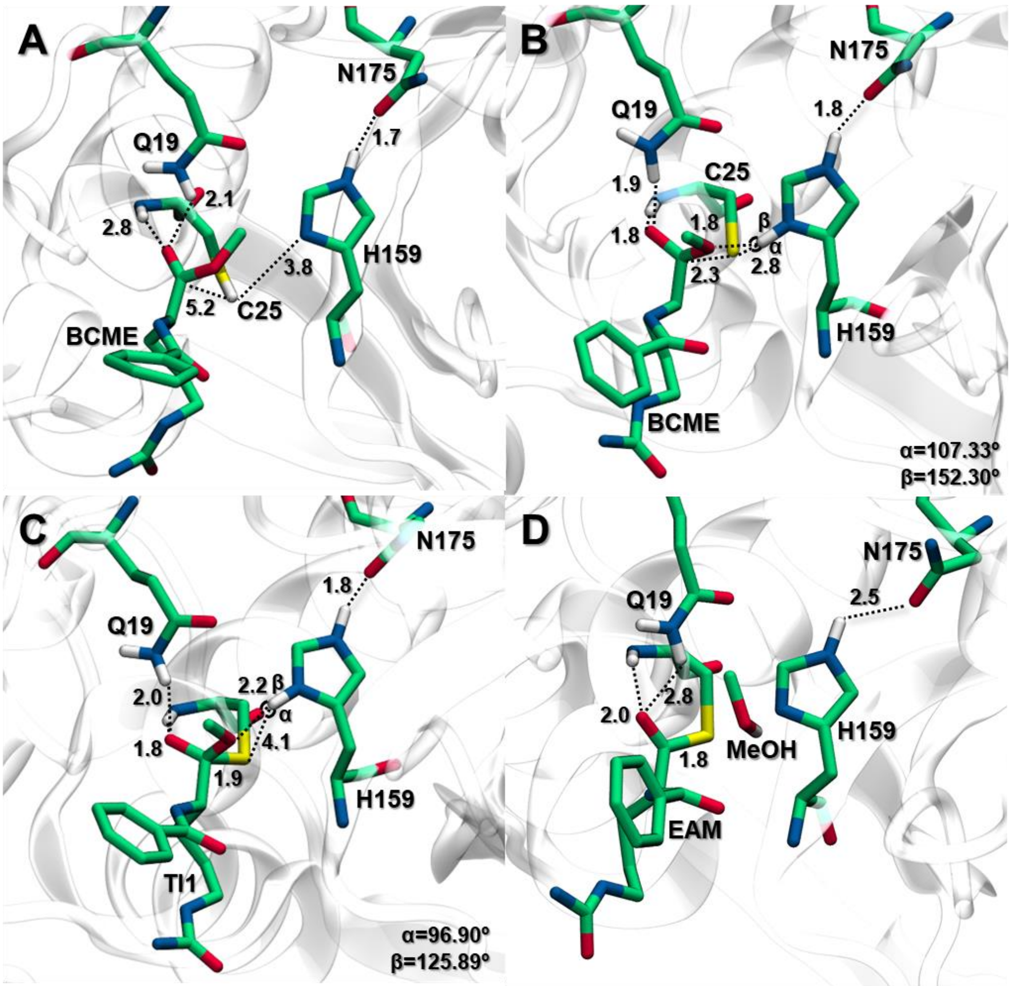

Figure S6. Active site pocket reference structures of the lowest-energy stationary points **RC_{SH}**, **RC_{S-}**, **TI1**, and **EAM** (**A** to **D**, respectively) of α-*N*-benzoyl-L-citrulline methyl ester (**BCME**). Key distances are given in Å, and the free methanol is shortened to **MeOH**.

Deacylation. The second part of the mechanism culminates in the release of the product complex **PC**, which is the α-*N*-benzoyl-L-citrulline (BC). This is achieved by the nucleophilic attack of a water molecule to the carbonyl carbon of the substrate (4.39 Å) and water proton transfer to the H159 (1.97 Å) to produce the **TI2**. In the **EAM** structure, there are at least two water molecules in the active site pocket that could perform the attack (Figure S7A). The energy barrier for this reaction (**TS3**) is 6.43 kcal mol^{-1}. Similar to the first tetrahedral intermediate, **TI2** presents substrate interaction with the oxyanion hole residues (Q19: 1.92 Å, and C25: 1.80 Å), stabilizing the negative charge of the substrate's oxygen atom (Figure S7B). The catalytic H159 is similarly close to the carboxylic acid oxygen of the substrate and the thiolate of C25 (2.28 Å *vs.* 2.55 Å); however, as **TI2** is endergonic concerning the **EAM**, the reaction will preferably follow the reverse direction. The reaction would continue to the products in a stepwise mechanism, with the release of BC. **PC_{S-}** has a free energy of 9.47 kcal mol^{-1},

which is 1.56 and 0.73 kcal mol^{-1} below **TS4a** and **TS4b**, respectively, transitioning to **PC$_{SH}$**, with 5.65 kcal mol^{-1} (Figure S7C,D).

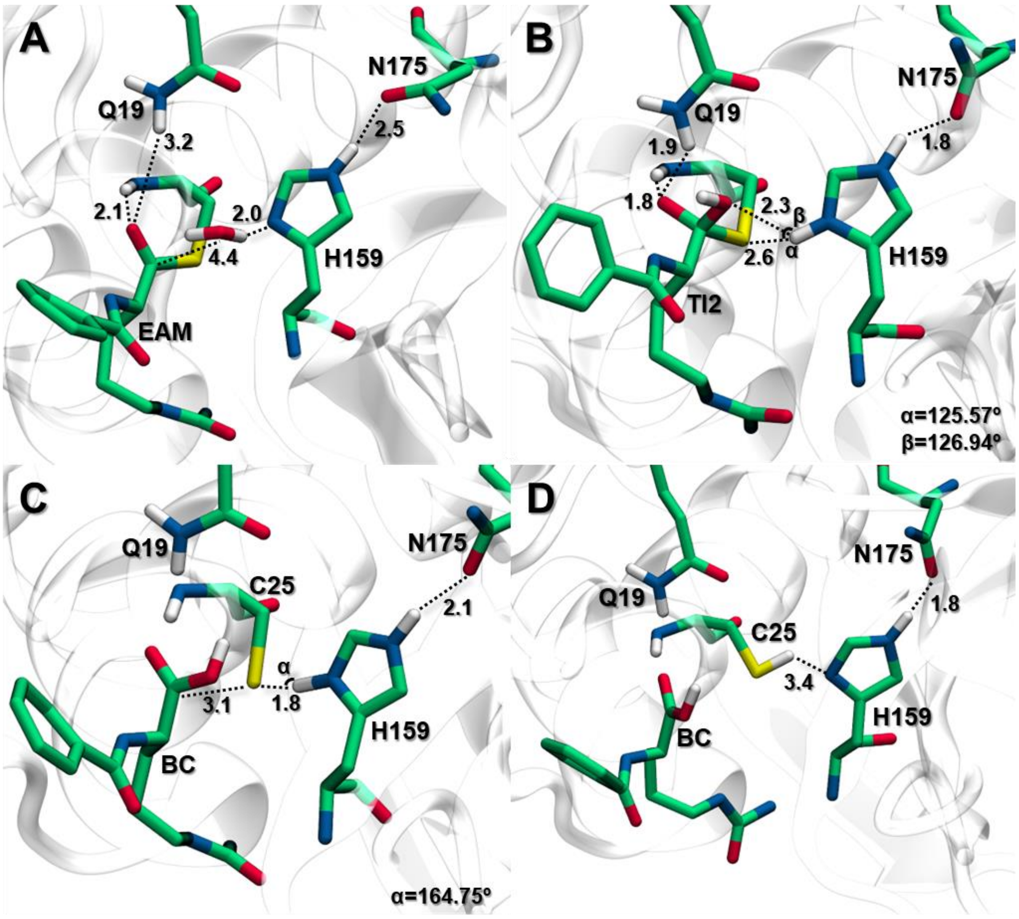

Figure S7. Active site pocket reference structures of the lowest-energy stationary points **EAM**, **TI2**, **PC$_{S-}$** and **PC$_{SH}$** (**A** to **D**, respectively) of α-*N*-benzoyl-L-citrulline methyl ester (**BCME**). Key distances are given in Å, and the product α-*N*-benzoyl-L-citrulline is shortened to **BC**.
